# Targeting Caspase-1 in osteoarthritis: multi-omics insights into the effects of VX-765 on human chondrocyte function and phenotype

**DOI:** 10.3389/fimmu.2025.1677801

**Published:** 2025-10-03

**Authors:** Jian Mei, Nicole Schäfer, Penghui Wei, Zhiheng Kong, Shushan Li, Patrick Pann, Marianne Ehrnsperger, Brian Johnstone, Eva Matalova, Susanne Grässel

**Affiliations:** ^1^ Department of Orthopedic Surgery, Experimental Orthopedics, Center for Medical Biotechnology (ZMB), Bio Park 1, University of Regensburg, Regensburg, Germany; ^2^ Department of Neurosurgery, Neurosurgery Research Institute, The First Affiliated Hospital of Fujian Medical University, Fuzhou, China; ^3^ Department of Neurosurgery, National Regional Medical Center, Binhai Campus of The First Affiliated Hospital, Fujian Medical University, Fuzhou, China; ^4^ Department of Orthopedic Surgery, The First Affiliated Hospital of Zhengzhou University, Zhengzhou, China; ^5^ Department of Trauma Surgery, University of Regensburg, Regensburg, Germany; ^6^ Department of Orthopedics and Rehabilitation, Oregon Health and Science University, Portland, OR, United States; ^7^ Institute of Animal Physiology and Genetics, Czech Academy of Sciences, Brno, Czechia; ^8^ Department of Physiology, University of Veterinary Sciences Brno, Brno, Czechia

**Keywords:** osteoarthritis, chondrocytes, caspase-1, VX-765, senescence, migration, MMP13, Mendelian randomization

## Abstract

**Background:**

Osteoarthritis (OA) progression involves chronic inflammation, chondrocyte senescence, and extracellular matrix (ECM) degradation affecting all synovial joint tissues. To date, no regenerative OA drugs have been approved. Caspase-1, a core effector of the inflammasome, may contribute to OA via both canonical inflammatory and non-canonical functions, but its therapeutic value remains unclear.

**Methods:**

We combined transcriptomic, proteomic, functional, and Mendelian randomization (MR) approaches. Using GSE168505 data, we analyzed CASP1, CARD gene family members (CARD16/17/18/8), and OA-related genes in OA- versus non-OA chondrocytes. We established an *in vitro* OA model by treating human chondrocytes with TNF-α ± VX-765 and assessed Caspase-1 activity, cell metabolism, and MMP secretion. We further conducted LC-MS/MS proteomic profiling, molecular docking, and MR analysis to identify molecular mechanisms and causal links.

**Results:**

CASP1 and inflammatory/ECM-degrading genes (e.g., IL1B, MMP13) were upregulated in OA chondrocytes, whereas SOX9 was downregulated. CASP1 gene expression correlated positive with genes involved in senescence, inflammation, oxidative stress and ECM remodeling. Inhibitor VX-765 significantly inhibited Caspase-1 activity, reduced senescence, and enhanced migration in non-OA- and OA chondrocytes, with donor-dependent effects in OA chondrocytes. It also suppressed MMP13 secretion in OA chondrocytes. Integrated transcriptomic and proteomic analysis showed that VX-765 reprogrammed OA-activated signaling, significantly downregulating pathways related to senescence, inflammation, complement activation, and ECM organization, while upregulating interferon-α/γ responses. Moreover, in silico performed molecular docking analyses suggest that caspase-1 may directly bind MMP13, CTSD, ABL1, MRPS11, POLR21, SMAD2 and SOX9. MR analysis supported a causal link between increased CARD17/18/8 gene expression and reduced OA risk; several CASP1 SNPs (e.g., rs61751523) showed negative OA associations, suggesting a protective role.

**Conclusions:**

This study demonstrates that Caspase-1 contributes to OA pathogenesis through both canonical and non-canonical mechanisms, and that VX-765 can alleviate chondrocyte dysfunction. The combined evidence supports VX-765 as a potential disease-modifying target for OA therapy. However, further investigation is warranted to clarify Caspase-1’s physiological roles, including possible off-target effects of its inhibitors, in cartilage and other joint tissues and the clinical relevance of inter-individual variability, with genomic variants (e.g., rs61751523) as one potential contributor, for therapeutic application.

## 1 Introduction

Osteoarthritis (OA) is a degenerative joint disease characterized by cartilage degradation, chondrocyte death, and chronic low-grade inflammation among other abnormalities (1). Despite its significant impact on patients’ quality of life, current clinical treatments primarily focus on symptomatic relief e.g. by application of nonsteroidal anti-inflammatory drugs (NSAIDs) and intra-articular injections (e.g., glucocorticoids and hyaluronic acid -HA). However, these interventions only provide short-term pain relief or functional improvement and fail to address the core pathological mechanism of OA—persistent inflammation driven by the innate immune system (2, 3). Additionally, while existing therapies provide immediate symptom relief, their long-term use can result in severe side effects. For instance, chronic use of NSAIDs is linked to gastrointestinal and cardiovascular risks, while repeated intra-articular injections of glucocorticoids have been associated with MRI-measured cartilage volume loss (4, 5). Accordingly, recent guidelines recommend intra-articular corticosteroids only conditionally, primarily for acute and short-term pain relief, and not for long-term or repeated use (6). Observational studies were reviewed to clarify if intra-articular HA or platelet-rich-plasma (PRP) injections (intervention) may delay the time to total knee arthroplasty (TKA) (outcome) among knee (K)OA patients (population), compared to KOA patients not receiving these injections (comparator). The results suggest that HA injections are associated with a 10-month delay in TKA in KOA. However, causality could not be concluded because of confounding factors and indication bias. Data were insufficient to conclude on any effect of PRP injections on TKA delay (7). Only very few emerging injectable biologics, including bone marrow aspirate concentrate (BMAC), show some promise in OA management (8). Therefore, the development of safe and effective disease-modifying OA drugs (DMOADs) targeting the fundamental disease mechanism is urgently needed.

Unlike rheumatoid arthritis (RA), a classic high-grade inflammatory arthritis, OA pathology involves a vicious cycle of chronic low-grade inflammation and impaired cartilage repair (9). Systemic or subcutaneous injections of existing RA biologics, such as anti-tumor necrosis factor (TNF) and anti-interleukin-1β (IL-1β), exhibit only limited efficacy in symptomatic OA and are ineffective in erosive OA subtypes (10). These disappointing clinical outcomes suggest that systemic targeting of single cytokines (e.g., IL-1β or TNF) may be insufficient for OA treatment (9).

Caspase-1, an effector of the NLRP3 inflammasome, is a promising therapeutic target in OA. It activates IL-1β and IL-18 and sustains chronic low-grade inflammation triggered by chondrocyte-derived danger signals (11–14).

Notably, beyond its canonical role in cytokine maturation, such as IL-1β activation, Caspase-1 also exerts non-canonical functions, including unconventional protein secretion and lysosomal regulation. These activities appear particularly relevant in non-immune cells, where Caspase-1 may contribute to stress responses (11). For instance, it regulates the release of actin-related proteins (e.g., ARPC1 and FGF2) (15), which may influence cartilage repair and tissue remodeling. More recently, studies have demonstrated additional roles of Caspase-1 in chondrogenesis, lipid metabolism, and bone remodeling. (16). However, its specific functions in OA development remain poorly understood.

Caspase-1 activity is tightly regulated through multiple mechanisms. For example, caspase activation and recruitment domain (CARD) proteins (e.g., CARD16, CARD17, CARD18) bind to pro-Caspase-1, preventing its interaction with the adaptor protein ASC (apoptosis-Associated speck-Like protein containing a CARD) and thereby inhibiting inflammasome- and Caspase-1 activation (17). Another key regulator, CARD8, exhibits dual roles: it suppresses Caspase-1 activation under normal conditions but promotes its activation upon cleavage or dissociation from dipeptidyl peptidase 8/9 (DPP8/9) (18). This bidirectional regulation presents both opportunities and challenges for Caspase-1-targeted therapies, particularly in balancing pathological inhibition with physiological function preservation (see **Supplementary Figure 1**).

Several peptidomimetic inhibitors (e.g., VX-740 and VX-765) have been developed to modulate Caspase-1 activity. These inhibitors selectively block Caspase-1 catalytic activity and demonstrate anti-inflammatory efficacy (11, 19). Although VX-740 showed promise in a Phase IIa trial for treatment of epilepsy, its development was halted due to hepatotoxicity (20). VX-765 has demonstrated therapeutic potential in preclinical models of neurodegenerative diseases (21, 22), yet its systemic or local application in OA pathology remains unexplored.

Despite advances in understanding Caspase-1’s role in NLRP3-driven OA pathology (23), critical knowledge gaps persist which are: (1) the spatiotemporal dynamics of Caspase-1 activity and its regulators (e.g., CARD8, CARD17/18) during OA progression; (2) potential off-target effects of systemic Caspase-1 inhibition on protein networks; and (3) whether Caspase-1 targeting can synergistically improve chondrocyte function and cartilage metabolism.

To this end, we propose the following hypothesis: Caspase-1 is abnormally expressed/activated in OA chondrocytes, and its pathological function may be driven by a specific DAMPs-NLRP3 signaling axis. Selective inhibition of Caspase-1 or induction of its negative regulators (such as CARD17/18) could potentially break the inflammation driven degradation cycle in OA. To validate this hypothesis, we integrated multi-omics analysis, genetic causal inference, and *in vitro* functional experiments, with the following aims: (1) to characterize the expression/activation patterns of Caspase-1 and its regulatory factors in chondrocytes; (2) to assess its potential as a target for intervention; and (3) to explore the impact of the Caspase-1 inhibitor VX-765 on chondrocyte functions (such as cell viability, proliferation, migration, and senescence) as well as on molecular network programming.

## 2 Materials and methods

### 2.1 Study design

**Figure 1:**
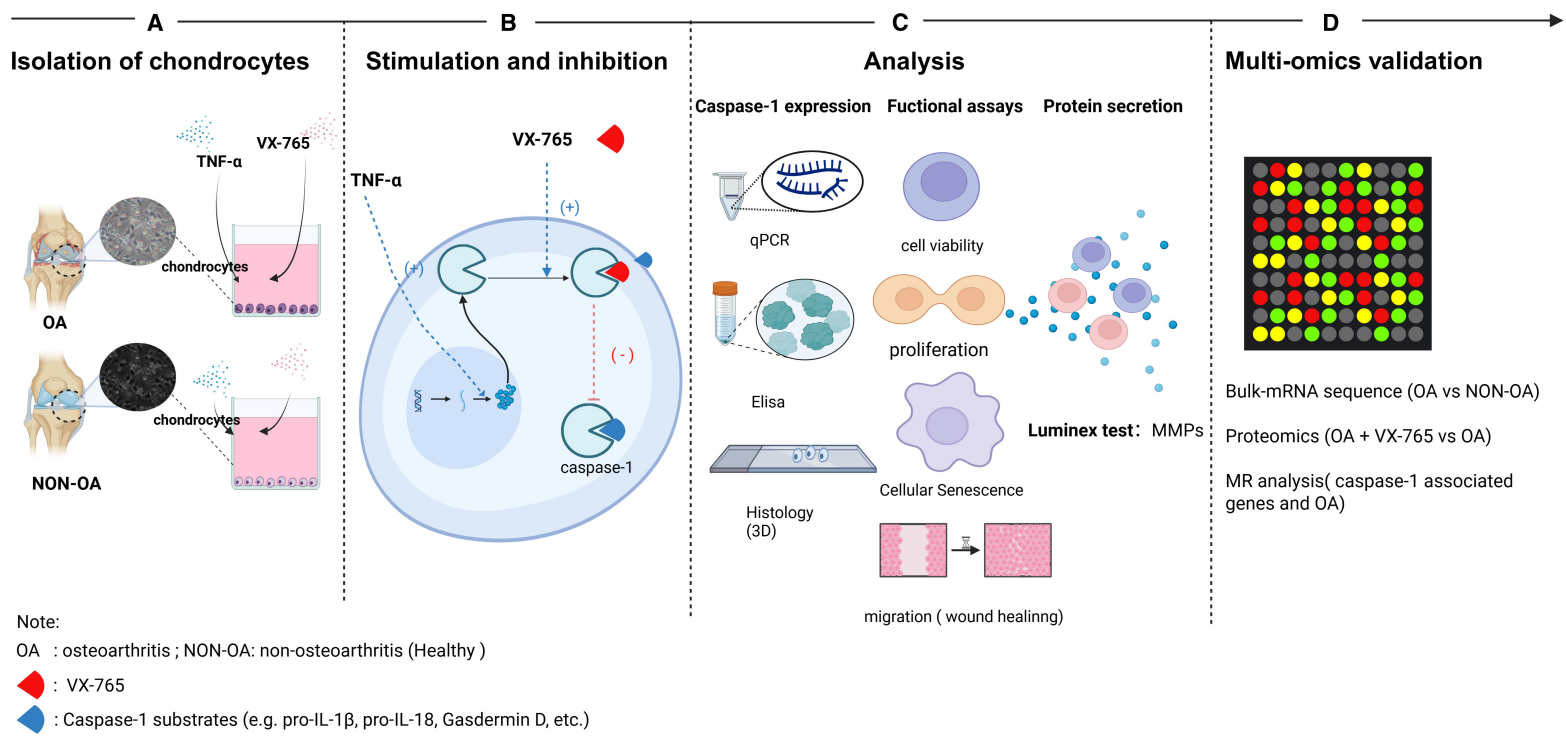
Schematic overview of the experimental workflow. **(A)** Isolation of primary chondrocytes: Articular cartilage was harvested from osteoarthritic (OA) and non-OA (healthy) human donors. Chondrocytes were enzymatically isolated and plated for subsequent assays. **(B)** Stimulation and inhibition: Cells were stimulated with TNF-α to promote Caspase-1 expression and co-treated with the Caspase-1 inhibitor VX-765. Arrows indicate Caspase-1 binding reactions (+) and their inhibition by VX-765 (–). **(C)** Phenotypic and molecular analyses: Caspase-1 expression was quantified by qPCR and ELISA; functional assays included cell viability, proliferation, senescence and migration and 3D IHC for caspase-1 localization. Secreted MMPs were measured by Luminex multiplex assays. **(D)** Multi-omics validation: Bulk RNA-seq. compared OA- vs. non-OA chondrocytes; proteomics contrastedOA ± VX-765; and Mendelian randomization (MR) assessed CASP1 genetic associations with OA. OA, osteoarthritis; NON-OA, non-osteoarthritic; VX-765, Caspase-1 inhibitor.

**Figure 1** presents the design framework of our study. We employed a triangulation methodology to comprehensively investigate the role of Caspase-1 in the pathogenesis of OA and its potential as a therapeutic target. First, we performed transcriptomic analyses using bulk RNA sequencing of public datasets to characterize the expression patterns of caspase-1 and related proteins in OA and non-OA chondrocytes. Second, we performed functional experiments using an *in vitro* OA model to assess the effects of the Caspase-1 inhibitor VX-765 on cellular behavior such as cell viability, proliferation, senescence, migration and MMP secretion. To further elucidate the downstream molecular mechanisms of Caspase-1 inhibition, we integrated transcriptomic and proteomic analyses, mapping key pathways and metabolic changes associated with VX-765 treatment. Third, we conducted genetic analyses, including genetic correlation and Mendelian randomization (MR), to evaluate the causal relationship between Caspase-1/CARD17/CARD18/CARD8 expression and OA risks. These combined analyses provide a comprehensive framework for better understanding the role of caspase-1 in OA pathogenesis and its therapeutic potential.

### 2.2 Human sample collection and ethical approval

Cartilage samples were collected from two groups: 17 OA patients undergoing elective total knee replacement surgery (Asklepiosklinikum, Bad Abbach, Germany) and 9 healthy individuals through cadaveric donations of femoral condyles (OHSU, Portland, OR, USA). Donors were enrolled consecutively on the basis of availability, and no stratification or balancing by age or sex was applied. The OA patients had an average age of 66.4 ± 7.2 years (ranging from 56 to 79 years), with 73.2% being female. In comparison, non-OA cartilage was obtained from cadavers, with an average age of 24 ± 6.3 years (ranging from 17 to 34 years) and 20.1% female donors. The use of human OA material has been approved by the local ethics committee (No. 22-2915-101; Ethikkommission, University of Regensburg, email: klinisches.ethikkomitee@klinik.uni-regensburg.de), and the written consent of all OA patients has been obtained.

### 2.3 Isolation and culture of chondrocytes

Chondrocytes were isolated as published previously (24). The cartilage tissue from OA donors was separated from the subchondral bone and cut into small pieces (~1–2 mm³) whereas the non-OA samples were received as a frozen aliquot of isolated chondrocytes (passage 0). The pieces were digested in type II collagenase (11 mg collagenase per 1 g cartilage) dissolved in Dulbecco’s Modified Eagle Medium (DMEM, #D8437, Sigma, UK) at 37 °C for 16 h. After digestion, the cell suspension was centrifuged at 300xg for 5 min. The chondrocytes were seeded into T175 culture flasks at a density of 5,000–10,000 cells/cm². The cells were expanded and cultured in DMEM F12 medium (Sigma Aldrich, Taufkirchen) supplemented with 10% fetal calf serum (FCS) (Sigma Aldrich, Taufkirchen) and 1% penicillin-streptomycin (Sigma Aldrich, Taufkirchen), with the medium replaced every 2–3 days. Once the cells reached 80–90% confluency, they were passaged. Passage 2–3 of all chondrocytes were used for the experiments.

### 2.4 *In vitro* OA low-grade inflammation model and VX-765 intervention

#### 2.4.1 Establishment of the OA low-grade inflammation model in 2D-monolayer

Human articular chondrocytes (both OA and non-OA) were seeded in 6-well plates at 1x10^4^ cells per well and treated with IL-1ß (0.5 or 1 ng/mL) or TNF-α (1 or 5 ng/mL) (Life Technologies, Darmstadt) for 24 h or 48 h (25). Untreated cells served as controls. After treatment, cells and supernatants were collected for subsequent analyses. At the experimental endpoint, Caspase-1 expression was assessed at both the mRNA and protein levels. Protein expression levels of Caspase-1 were quantified using enzyme-linked immunosorbent assay (ELISA) kits, with analyses performed on cell lysates.

#### 2.4.2 3D micromass pellets

Chondrocytes were trypsinized, counted, and resuspended at 1x10^5^ cells per pellet in complete chondrogenic medium composed of high-glucose DMEM (Life Technologies, Darmstadt) supplemented with ITS+ (Corning, Amsterdam), penicillin/streptomycin, 100 nM dexamethasone (Sigma Aldrich, Taufkirchen, Germany), 10 ng/mL TGF-β3 (Life Technologies, Darmstadt), 50 µg/mL ascorbate-2-phosphate, 40 µg/mL L-proline, and 1 mM sodium pyruvate (Sigma Aldrich, Taufkirchen, Germany) (26). Cell suspensions (240 µL) were seeded into 96-well conical-bottom plates, centrifuged at 500xg for 5 min, and incubated at 37 °C with 5% CO_2_ for 14 days with medium changes every other day. To induce an inflammatory response, TNF-α (final concentration 1 ng/mL) was added on day 12 (48-hour group), and pellets were harvested on day 14. Unstimulated controls were cultured in parallel without TNF-α.

#### 2.4.3 VX-765 intervention

Chondrocytes were treated with VX-765 (Invivogen, France) at a working concentration of 100 μM (50 μg/mL) for a total of 72 h. We dissolved VX-765 in DMSO to prepare a 10 mg/mL stock solution and diluted it in culture medium to achieve the desired concentration of 50 µg/ml. We pre-treated the chondrocytes with VX-765 for 24 h and then co-treated them with 1 ng/mL TNF-α for the subsequent 48 h to assess functional effects.

### 2.5 PCR detection

#### 2.5.1 Primers

Gene-specific primer pairs were designed using NCBI Primer-BLAST and synthesized by Microsynth, Switzerland (HPLC purification grade). Primer sequences with corresponding references are summarized in **Table 1**.

**Table 1:** Primer sequences for target and reference genes.

Gene	Forward primer (5’-3’)	Reverse primer (5’-3’)
Caspase-1	GCTGTACGAGAAGGTCATCC	TCCAGTCAGAAAGGTGCTGA
HPRT1	TGACACTGGCAAAACAATGCA	GGTCCTTTTCACCAGCAAGCT
TFRC	CAGTCACCGTGTCTTCCTCA	GCTGCCTTCAAACTCATCCG

#### 2.5.2 RNA extraction and qRT-PCR analysis

Total RNA was extracted from chondrocytes using the RNeasy Mini Kit (Qiagen, Cat. No. 74104) according to the manufacturer’s instructions. RNA purity was assessed by measuring the absorbance ratio at 260/280 nm (acceptable range: 1.8–2.0), and RNA concentration was determined using a spectrophotometer (Thermo Scientific, Darmstadt, Germany). Equal amounts of RNA were used for reverse transcription across all samples to ensure standardized cDNA synthesis. Complementary DNA (cDNA) was synthesized using the AffinityScript QPCR cDNA Synthesis Kit (Agilent Technologies, Waldbronn, Germany) following the manufacturer’s protocol. Quantitative real-time PCR (qRT-PCR) was then performed using Brilliant III Ultra Fast SYBR QPCR (Agilent Technologies, Waldbronn) on an MX3005P QPCR System (Agilent Technologies, USA). The PCR cycling conditions were as follows: initial denaturation at 95 °C for 2 min, followed by 40 cycles of denaturation at 95 °C for 5 s and annealing/extension at 60 °C for 10 s. Relative gene expression levels were calculated using the ΔΔCt method and normalized to HPRT1 and TFRC as internal reference genes. All qRT-PCR reactions were carried out in duplicate.

### 2.6 ELISA detection

Caspase-1 protein levels were quantified using two commercially available ELISA kits: the Quantikine™ Human Caspase-1/ICE Immunoassay (R&D Systems, Cat. No. DCA100) and the RayBio^®^ Human Caspase-1 ELISA Kit (RayBiotech, Cat. No. ELH-Caspase1), following the manufacturers’ protocols. All samples were measured in duplicate, and absorbance was recorded at 450 nm using a microplate reader (Molecular Devices, Munich, Germany). Concentrations were calculated from standard curves generated by using a four-parameter logistic (4-PL) model.

### 2.7 Cell function assays and MMPs secretion quantification

#### 2.7.1 Caspase-1 activity identification

Caspase-1 activity was measured using the Caspase-Glo^®^ 1 Inflammasome Assay (Promega, Walldorf, Cat. #G9951). 1x10^4^ cells were seeded in white, opaque-walled 96-well plates (Thermo Scientific, Nunc™, Cat. #136101) and left 24 h for attachment. Following the experimental treatments, 100 μL of the Caspase-Glo^®^ 1 Reagent, prepared by reconstituting the Z-WEHD substrate in the supplied buffer with MG-132, was added directly to each well containing 100 μL of cell culture medium. Plates were gently mixed and incubated at room temperature for 1 h to allow luminescent signal stabilization. Luminescence was measured using a microplate luminometer (Molecular Devices, Munich), and relative luminescence units (RLUs) were recorded as a readout of Caspase-1 activity. As control we included DMSO (untreated cells), and blank reactions were performed to subtract background luminescence.

#### 2.7.2 Cell viability

Cell viability was assessed using the CellTiter-Blue^®^ Cell Viability Assay (Promega, Cat. No. G8081). Chondrocytes were seeded in 96-well plates at a density of 1x10^4^ cells per well in 100 µL of DMEM/F12 medium supplemented with 10% FCS and 1% penicillin-streptomycin. After leaving cells for 24 h to attach, cells were treated according to the experimental conditions, including DMSO (control without inhibitor), TNF-α (1 ng/mL) stimulation and/or Caspase-1 inhibition using VX-765 (100 µM). At the end of the treatment, 20 µL of CellTiter-Blue^®^ reagent was added directly to each well. The plates were gently shaken for 10 s to ensure uniform distribution of the reagent and incubated at 37 °C for 4 h. After incubation, absorbance was determined on a microplate reader (Molecular Devices, Munich, Germany) at 570 nm (test wavelength) and 600 nm (reference wavelength). Background absorbance (wells containing medium and reagent only) was subtracted, and each condition was assayed in duplicate.

#### 2.7.3 Cell proliferation

Cell proliferation was assessed using a BrdU ELISA kit (Roche, Cat. No. 11647229001). Chondrocytes were seeded into 96-well plates at a density of 1x 10^4^ cells per well in 100 µL of DMEM/F12 medium supplemented with 10% FCS and 1% penicillin-streptomycin. After 24 h cell attachment, the cultures were treated with DMSO (control without inhibitor), TNF-α (1 ng/mL) and/or VX-765 (100 µM) for 72 h. On the final day, BrdU labeling solution (10 µL/well; final concentration: 10 µM) was added and cells were incubated at 37 °C for 24 h. After incubation, the cells were fixed and DNA was denatured by adding 200 µL of FixDenat solution per well for 30 min at room temperature. Subsequently, 100 µL of anti-BrdU-POD antibody working solution (1:100 dilution) was added, and the plate was incubated for 90 min. After three washes with PBS, 100 µL of TMB substrate solution was added to each well and allowed to develop until sufficient color was observed. The enzymatic reaction was stopped by adding 25 µL of 1 M H_2_SO_2_. Absorbance was measured at 450 nm with a reference wavelength of 690 nm using a microplate reader (Molecular Devices, Munich, Germany). All samples were analyzed in triplicate.

#### 2.7.4 Cellular senescence assay

We evaluated cellular senescence using the Senescence-Associated (SA)-β-Gal Activity Assay Kit (Cell Biolabs, Cat. No. CBA231) according to the manufacturer’s instructions. Chondrocytes were seeded into 96-well plates at a density of 1x10^4^ cells per well and cultured in DMEM/F12 medium supplemented with 10% FCS and 1% penicillin-streptomycin. After the experimental treatments, cells were washed once with cold PBS (200 µL/well) and lysed with 50 µL of cold 1x Lysis Buffer containing protease inhibitors. The plate was incubated on ice for 5 min, followed by centrifugation at 4 °C (3,000xg for 10 min). For the reaction setup, 10 µL of the supernatant from each well was transferred to a U-bottom black 96-well plate, and 10 µL of 2x Reaction Buffer containing β-mercaptoethanol and SA-β-Gal substrate was added to each sample. The plate was incubated at 37°C for 1 h in the dark. The reaction was stopped by adding 100 µL of Stop Solution per well. Fluorescence intensity was measured using a microplate reader (Molecular Devices, Munich, Germany) at an excitation wavelength of 360 nm and an emission wavelength of 465 nm. All conditions were performed in triplicate.

#### 2.7.5 Migration

Chondrocyte migration was evaluated using a wound healing (scratch) assay with ibidi culture inserts (Cat. No. 80206, ibidi, Germany). Cells were seeded into the two chambers of each insert at a density of 2x10^4^ cells per chamber in DMEM/F12 medium supplemented with 10% fetal calf serum (FCS) and 1% penicillin-streptomycin. After 24–48 h of incubation, cells reached full confluence. The inserts were then gently removed to create a defined cell-free gap. Wells were washed twice with PBS to remove non-adherent cells and debris (27). Cells were subsequently treated with DMSO (control without inhibitor), TNF-α (1 ng/mL) and/or VX-765 (100 µM) diluted in DMEM/F12 medium containing 1% FCS. Images of the gap area were captured using a phase-contrast microscope (Eclipse TS100, Nikon, Japan) at 0, 24, 48, 72, and 96 h post-treatment. Gap closure, indicative of cell migration, was quantified using ImageJ software (version 1.8.0, National Institutes of Health, USA). All conditions were performed in duplicate.

#### 2.7.6 MMPs quantification by luminex multiplex assay

Matrix metalloproteinases (MMPs) in cell culture supernatants were quantified using the PROCARTAPLEX 6 PLEX Human MMP Panel (ThermoFisher Scientific, USA) on a Bio-Plex^®^ 200 system (Bio-Rad Laboratories, USA). The assay was performed according to the manufacturer’s instructions, utilizing bead-conjugated capture antibodies, biotinylated detection antibodies, and streptavidin-PE for signal detection. Supernatants were collected and stored at −80°C. Undiluted samples (50 µL) were analyzed in single wells. Background signals were subtracted using cell-free control wells. Data were acquired and analyzed using Bio-Plex Manager™ software (Bio-Rad Laboratories, USA) with five-parameter logistic (5-PL) regression.

### 2.8 Downstream analyses of 3D chondrogenic pellets

#### 2.8.1 Pellet fixation and paraffin embedding

After completion of the 3D chondrogenic culture at 2 weeks, pellets were gently harvested and transferred into 2 mL microcentrifuge tubes. Samples were briefly washed with PBS and fixed in 1 mL of freshly prepared 4% paraformaldehyde (Merck, Darmstadt, Germany) (PFA in PBS, pH 7.4) overnight at 4 °C (not exceeding 24 h). After fixation, pellets were rinsed in PBS and dehydrated through a graded ethanol series: 50% ethanol (Walter CMP, Kiel, Germany) (2–6 h), 70% ethanol (2–6 h, with optional long-term storage at 4°C), followed by 96% ethanol (2–6 h twice, with the second incubation overnight). Subsequently, pellets were incubated in isopropanol (Walter CMP, Kiel) (2–6 h twice), then transferred to a 1:1 mixture of isopropanol and paraffin at 60°C for 24 h. Final embedding was performed via two immersions in 100% paraffin at 60°C for 24 h and 12 h, respectively. Paraffin blocks were processed for microtome (Leica, Wetzlar, Germany) sectioning.

#### 2.8.2 RNA extraction

3D chondrocyte pellets were rinsed twice with PBS and transferred to 2 mL RNase-free tubes. Pellets were lysed in 600 µL Tissue and Cell Lysis Solution (MasterPure™ Complete RNA Purification Kit, Epicentre, Cat. No. MC85200) supplemented with 2 µL Proteinase K. Samples were incubated at 65 °C for 2x5 min and 1x3 min with intermittent vortexing and gentle compression using RNase-free pestles to ensure complete disruption. Lysates were then chilled on ice for 5–10 min. Proteins were precipitated by adding 300 µL MPC Protein Precipitation Reagent, vortexing for 10 s, and centrifuging at 13,000xg for 10 min at 4°C. The supernatant was transferred to a fresh tube, and total nucleic acids were precipitated with 1 mL ice-cold isopropanol by 30–40 inversions, followed by centrifugation at 10,000xg for 10 min at 4°C. The supernatant was discarded, and the pellet was resuspended in 200 µL of DNase master mix (195 µL 1x DNase buffer + 5 µL RNase-free DNase I), then incubated at 37°C for 20 min. After DNase treatment, 200 µL 2x Tissue and Cell Lysis Solution and 200 µL MPC Protein Precipitation Reagent were added sequentially, with brief vortexing and a 5–10 min ice incubation, then centrifuged again at 13,000xg for 10 min (4°C). The cleared supernatant was transferred to a new tube, and RNA was re-precipitated with 500 µL ice-cold isopropanol (30–40 inversions), followed by centrifugation at 10,000xg for 10 min at 4°C. The RNA pellet was washed twice with 500 µL 70% ethanol (centrifugation at 10,000 x g, 3 min, 4°C), air-dried under a laminar-flow hood for 5–10 min, then resuspended in 30–50 µL RNase-free water. RNA concentration and purity were determined by NanoDrop™ spectrophotometry (Thermo Scientific, Darmstadt).

#### 2.8.3 Protein extraction

For total protein analysis, cell pellets were washed in ice-cold PBS and lysed in RIPA buffer (50 mM Tris-HCl, 150 mM NaCl, 1% NP-40, 0.5% sodium deoxycholate, 0.1% SDS, pH 7.4) supplemented with protease and phosphatase inhibitor cocktails (Roche, Cat. Nos. 04693132001 and 04906837001). Samples were homogenized by repeated pipetting and vortexing, followed by centrifugation at 12,000xg for 15 min at 4 °C. The supernatant was collected and stored at −80 °C until further use.

#### 2.8.4 Immunohistochemistry and Alcian Blue co-staining

5 µm thick sections were prepared from Paraffin-embedded micromass pellets and mounted on Superfrost™ Plus slides (Englbrecht, Edermünde, Germany). Sections were deparaffinized in Rotihistol (Carl Roth, Karlsruhe, Germany) (2 x 15 min) and rehydrated through a graded ethanol series (10 min each in 99%, 96%, 70%, and 50%) followed by distilled water. Antigen retrieval was performed in citrate buffer (pH 6.0) at 60 °C for 23–24 h, followed by cooling at room temperature. Endogenous peroxidase activity was blocked using 3% hydrogen peroxide (Carl Roth, Karlsruhe) for 10 min. After washing with TBS + 0.1% Tween-20 (2 × 7 min), sections were blocked with 5% goat serum (Biozol, Hessisch Oldendorf, Germany) in TBS-T for 1 h at room temperature. Primary antibodies—anti-cleaved caspase-1 p20 (Thermo Fisher Scientific, Cat. No. PA5-99390, used at a dilution of 1:200), anti-caspase-1 (Thermo Fisher Scientific, Cat. No. PA5-119002, used at a dilution of 1:250), and rabbit IgG isotype control (Novus Biologicals, Cat. No. NBP1-97041)—were diluted in SignalStain^®^ Antibody Diluent (Cell Signaling Technology, Cat. No. 8112) and applied at 50 µL per section overnight at 4°C in a humidified chamber. The next day, sections were incubated for 30 min at room temperature with SignalStain^®^ Boost IHC Detection Reagent (HRP, Rabbit). Signal was developed using a DAB solution (1.25 mL DAB + 1 µL 30% H_2_O_2_, 50 µL per section) for 1–15 min depending on staining intensity. Nuclear counterstaining was performed using Gill III hematoxylin (Merck, Darmstadt) for 1 s, followed by sequential rinses in distilled water (10x, 20x), 0.1% HCl (Carl Roth, Karlsruhe) (5x), and water. For Alcian Blue (Serva, Heidelberg) co-staining, sections were treated with 3% acetic acid (Carl Roth, Karlsruhe) (pH 2.5) (pH 2.5) for 3 min and then incubated for 30 min in 1% Alcian Blue (prepared in 3% acetic acid). Slides were rinsed in tap water (10 min) and distilled water (5 min), dehydrated through ascending ethanol concentrations (50%, 70%, 96%, and 99%, 5 min each), cleared in Rotihistol (2x10 min), and mounted using Rotihistokitt (Carl Roth, Karlsruhe). Slides were dried under a fume hood for 24 h before imaging with a microscope (Nikon Europe, Amstelveen, Netherlands).

### 2.9 Public database and bioinformatics analysis

Bulk RNA-seq data from OA- and non-OA chondrocytes were analyzed using publicly available datasets from the Gene Expression Omnibus (GEO, GSE168505). Raw count data were pre-processed and normalized using DESeq2 in R (v4.2.2). Low-count genes were filtered out, and normalization was performed using the median ratio method. Differentially expressed genes (DEGs) were identified with an adjusted p-value (padj) < 0.05 and |log2FoldChange| > 0.9. Functional enrichment analyses included over-representation analysis (ORA) and gene set enrichment analysis (GSEA). ORA was conducted on significantly upregulated and downregulated DEGs using the clusterProfiler package, focusing on Gene Ontology (GO) terms and Kyoto Encyclopedia of Genes and Genomes (KEGG) pathways, with results visualized as dot plots and bar plots. GSEA was performed on the ranked gene list using the fgsea package, with enrichment scores calculated for MSigDB Hallmark gene sets and SAUL_SEN_MAYO gene set. Gene sets with padj < 0.05 were considered significant, and results were visualized as enrichment plots. DEGs were visualized using volcano plots generated with ggplot2 and EnhancedVolcano, highlighting Caspase-1 and associated genes (e.g., CARD17). Transcripts per Million (TPM) values were used to compare caspase gene expression between OA and non-OA groups, with results shown as bar plots. All analyses were performed using R and associated packages. For methods on gene expression correlation analyses, protein–protein interaction network construction, hub-gene screening, and molecular docking analysis, please refer to the **Supplementary Methods**.

### 2.10 Mendelian randomization analysis

We conducted two-sample MR analyses to explore the causal effects of multiple caspase-related genes (e.g., CASP1, CARD8, CARD17, CARD18) on the risk of OA. Single-nucleotide polymorphisms (SNPs) significantly associated with gene expression (p < 5x10^-8^) were extracted as instrumental variables from European ancestry eQTL datasets. Linkage disequilibrium (LD) clumping was applied (window size = 100 kb, r² < 0.3), and SNPs with minor allele frequency (MAF) ≤ 0.01 were excluded. Harmonized exposure and outcome datasets were analyzed using the inverse-variance weighted (IVW) method to estimate the primary causal effects. Sensitivity analyses included MR-Egger regression, weighted median estimation, single-SNP analysis. Horizontal pleiotropy and heterogeneity were assessed using the MR-Egger intercept and Cochran’s Q statistic, respectively. All statistical analyses and visualizations were performed using the TwoSampleMR R package (v0.5.6). Further details of gene-specific SNP selection, genomic windows, and the analysis pipeline are provided in the **Supplementary Methods**.

### 2.11 Proteomics analysis

Proteins were extracted from chondrocytes treated with VX-765 (100 µM) or DMSO (vehicle control) on the third day of treatment, samples (three VX 765–treated and three vehicle controls) were transferred to 1.5 mL microcentrifuge tubes and lysed in DB buffer (denaturing buffer; 6 M urea, 100 mM triethylammonium bicarbonate [TEAB], pH 8.5) by sonication on ice for 5 min, then clarified by centrifugation at 12 000xg for 15 min at 4°C. The supernatant was reduced with 100 mM DTT at 56°C for 1 h and alkylated with an excess of iodoacetamide for 1 h at room temperature in the dark, followed by a 2 min ice bath. Samples were diluted to <1 M urea with 100 mM TEAB and digested overnight at 37°C with sequencing grade trypsin (1:50, w/w). Peptides were acidified, desalted on C18 cartridges (Thermo Fisher Scientific), and dried by vacuum centrifugation. LC MS/MS analysis was performed on an Orbitrap Astral HRAM mass spectrometer (Thermo Fisher Scientific) coupled to nano LC: peptides were separated on a 75 μm x 25 cm C18 column using a 5–30% acetonitrile gradient in 0.1% formic acid at 300 nL/min. DIA (data-independent acquisition) comprised a full scan MS (m/z 40–6000, resolution 80–000 at m/z 524) followed by sequential MS2 windows (resolution >50–000 at m/z 130) at up to 200 Hz with single microscan. Raw data were processed via a library free workflow (e.g. Spectronaut or DIA NN) against the UniProt proteome, with peptide and protein level identifications filtered at 1% FDR (false discovery rate) and label free quantification based on MS2 fragment ion peak areas. Subsequent proteomic analyses—including differential protein identification, pathway enrichment, PPI network construction, and molecular docking—are described in detail in the **Supplementary Methods**.

### 2.12 Statistical assumptions and software

All analyses assumed approximate normality, homogeneity of variances, and independence of observations, with paired designs applied for within-donor comparisons. RNA-seq analyses were conducted in R using DESeq2, clusterProfiler, enrichplot, fgsea, GseaVis, ggplot2, and pheatmap. Proteomic data were processed with DIA-NN, with downstream analyses in R using the same packages and the Metascape platform. PPI networks were retrieved from STRING and analyzed in R with igraph, tidygraph, and ggraph. Molecular docking was performed using PyMOL, GRAMM-X, and PDBePISA. Mendelian randomization analyses were conducted in R with the TwoSampleMR package, and PheWAS/motif disruption analyses were performed on the MSK-HuGEAMP platform.

### 2.13 Statistical analysis

All statistical analyses were conducted in GraphPad Prism (v10.2.3) or R (v4.2.2). Data are presented as median ± interquartile range (IQR). Caspase-1 gene expression was calculated as log2-fold change relative to the untreated control (set to zero) and tested by one-sample t-test. Two‐group comparisons (e.g. treated *vs*. control within the same donor) used paired t-tests. When more than two groups were compared (control, +TNF-α, +VX-765, +TNF-α + VX-765), differences were assessed by one-way ANOVA followed by Holm–Sidak *post hoc* tests or two-way ANOVA with Geisser-Greenhouse correction followed by Šídák’s multiple comparisons test (as detailed in the figure legends). All tests were two-tailed, and P < 0.05 was considered statistically significant.

## 3 Results

### 3.1 Comparison of Caspase-1 expression in OA- versus non-OA chondrocytes

**Figure 2:**
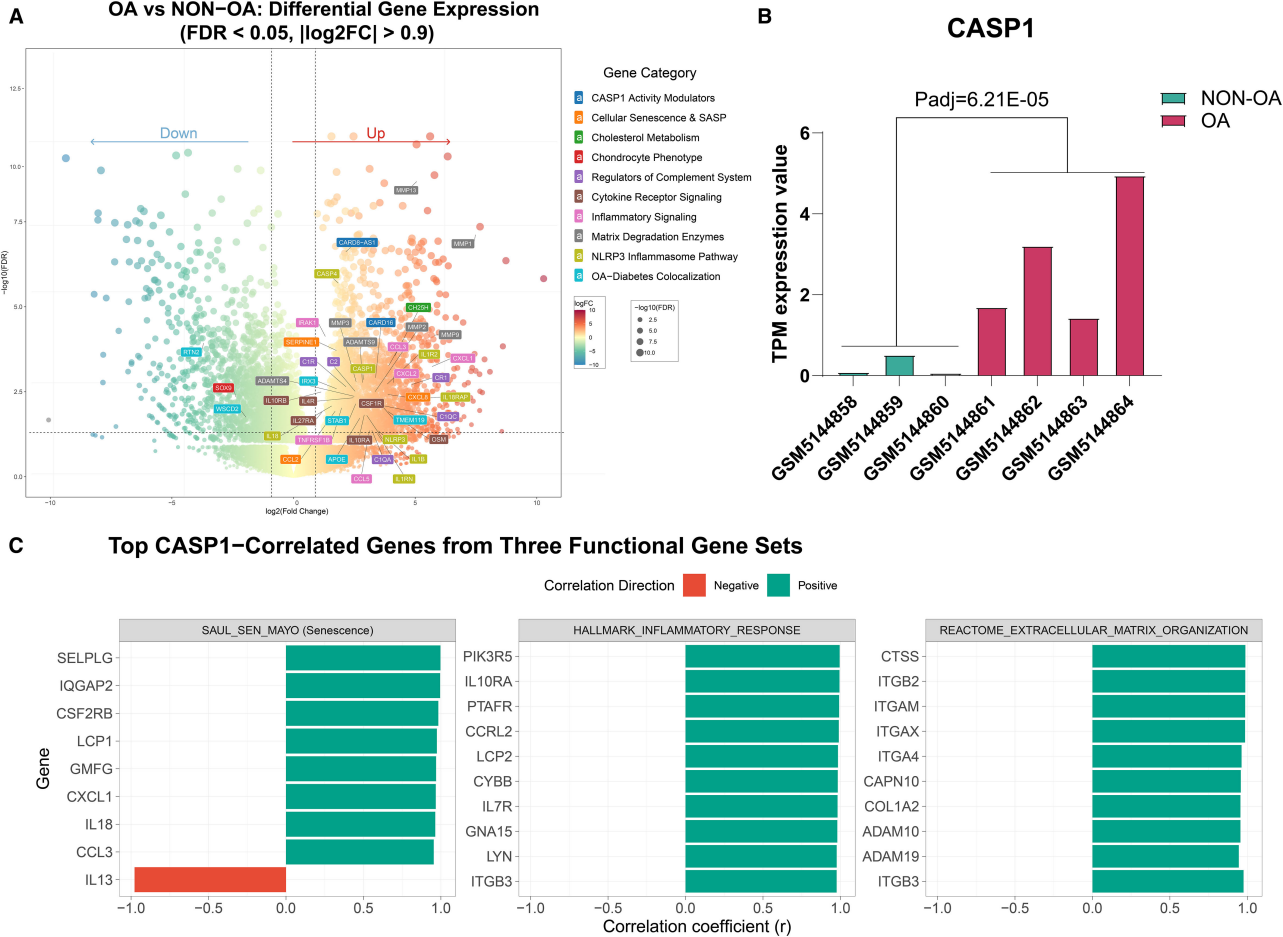
Differential gene expression and correlation analysis of CASP1 in OA- and non-OA chondrocytes based on the GEO dataset (GSE168505). **(A)** Volcano plot illustrating differentially expressed genes between OA- and non-OA cartilage samples (FDR < 0.05, |log_2_FC| > 0.9). **(B)** Bar plot showing CASP1 gene expression (transcripts per million = TPM values) across five independent datasets, consistently upregulated in OA samples compared to non-OA (Padj = 6.21E−05). **(C)** Pearson correlation analysis between CASP1 expression and gene sets from selected pathways. For each pathway, the top 10 genes most strongly correlated with CASP1 are shown. Red bars indicate negative correlations and green bars indicate positive correlations. All displayed correlations are statistically significant (FDR < 0.05); the exact FDR (q) values are provided in **Supplementary Table S1**.

To characterize the expression pattern of Caspase-1 in OA pathogenesis, we analyzed publicly available bulk RNA-sequencing data (GSE16850**5**) comparing OA and non-OA chondrocytes. CASP1 expression was significantly upregulated in OA chondrocytes and showed consistent elevation across all samples (**Figures 2A, B**). This upregulation was accompanied by increased expression of its homolog CASP4, the inflammasome component NLRP3, and downstream effectors IL-1β and IL-18. Regulatory factors, including CARD16 and the antisense transcript CARD8-AS1, were also markedly elevated (**Figure 2A**).

Additionally, potential Caspase-1-responsive genes related to complement system activation, lipid metabolism, and other associated pathways showed coordinated upregulation (**Figure 2A**). Correlation analysis further revealed that CASP1 expression was significantly associated with gene sets linked to cellular senescence, inflammatory response, oxidative stress, extracellular matrix remodeling, and cell cycle regulation. (**Figure 2C**, **Supplementary Table S1**).

These results show that OA chondrocytes exhibit dysregulation of Caspase-1 expression and its regulatory network, suggesting coordinated activation of the inflammasome pathway in the disease state.

### 3.2 TNF-α dose-dependent effects on Caspase-1 expression in chondrocytes

To establish a chronic low-grade inflammatory model, we tested the effects of IL-1β and TNF-α on Caspase-1 expression in OA- and non-OA chondrocytes cultured as 2D monolayers and 3D micromass pellets. IL-1β stimulation did not significantly alter Caspase-1 expression at either the gene nor protein level (**Supplementary Figure 2**).

**Figure 3:**
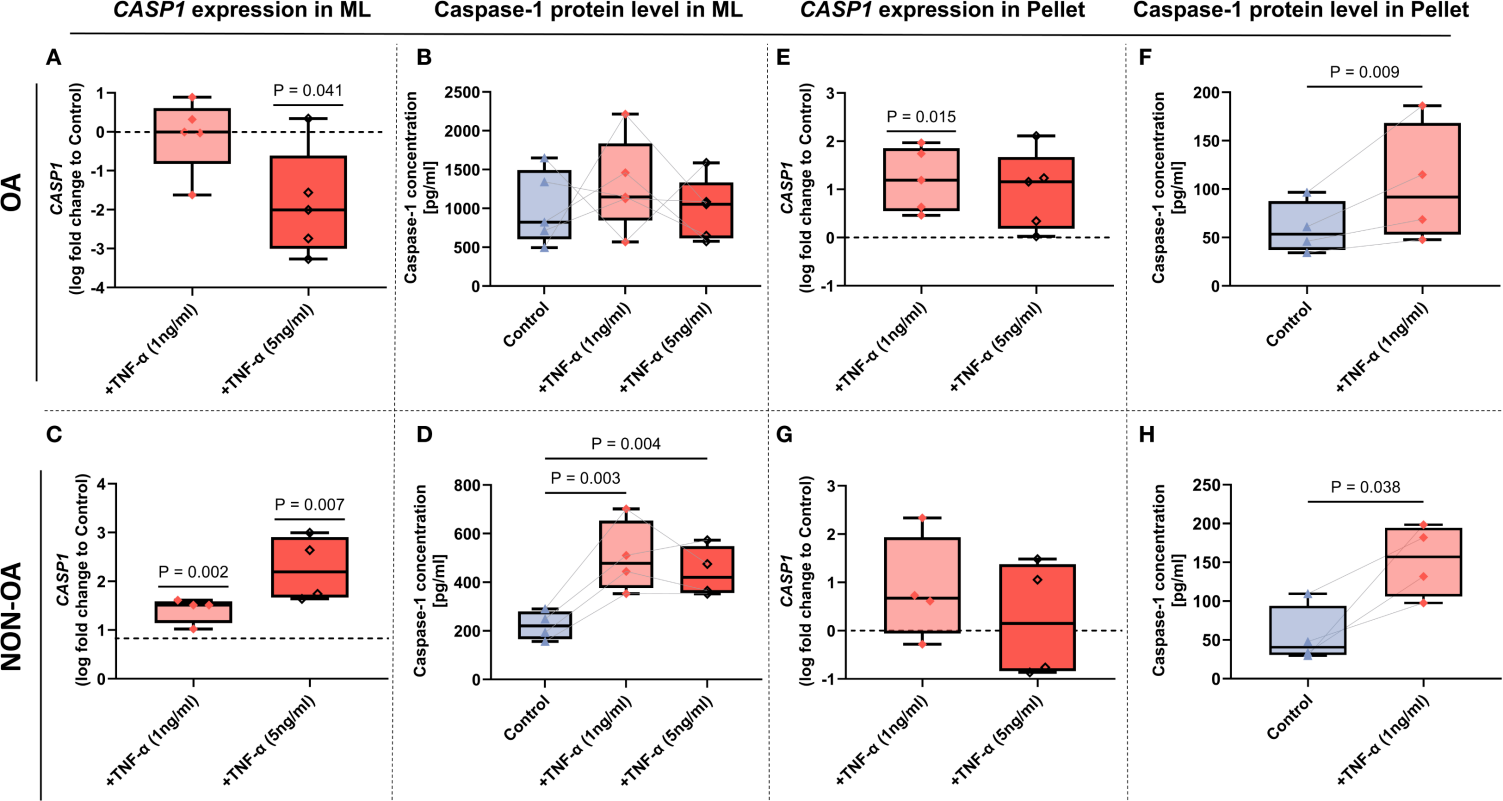
Caspase-1 mRNA and protein expression in OA- and non‐OA chondrocytes under TNF-α stimulation in monolayer and micromass pellet culture systems. **(A–D)** Monolayer (2D) cultures. CASP1 mRNA expression in OA- **(A)** and non-OA **(C)** chondrocytes following 24 hour TNF-α stimulation (1 and 5 ng/mL), presented as log_2_-fold change relative to unstimulated controls. Caspase-1 protein in cell lysates of OA- **(B)** and non-OA **(D)** cultures is expressed in pg/ml. **(E–H)** Micromass pellet (3D) cultures. CASP1 mRNA expression in OA- **(E)** and non-OA **(G)** chondrocyte pellets after TNF-α (1 and 5ng/mL) treatment for 24 hours, shown as log_2_-fold change versus unstimulated pellets. Corresponding Caspase-1 protein in lysates of OA- **(F)** and non-OA **(H)** pellets after TNF-α (1 ng/ml) treatment, expressed in pg/ml. Box plots show median (horizontal line), interquartile range (box), and individual data points. Gene expression was evaluated by one-sample t-tests (N = 4–5 independent experiments); cell-lysate protein measurements were analyzed by one-way ANOVA with Holm–Sidak *post hoc* tests **(B, D)** or Paired t-test **(F, H)** (N = 4–5). P-values < 0.05 were considered significant.

In contrast, TNF-α stimulation led to a more consistent and significant upregulation of Caspase-1 at both the transcriptional and protein levels in OA- and non-OA chondrocytes. Under stimulation with 1 ng/mL TNF-α, Caspase-1 expression exhibited a robust and mostly consistent induction upward trend (**Figure 3**) – an effect that was notably more pronounced in 3D pellet cultures (**Figures 3E-H**) than TNF-α stimulation in 2D monolayers (**Figures 3A-D**).

Notably, extended stimulation (>72 hours) (not shown) or higher TNF-α concentrations (5 ng/mL) failed to further enhance Caspase-1 expression (**Figures 3A–H**).

Contrary, under high-dose TNF-α stimulation (5 ng/mL), CASP1 mRNA levels were significantly downregulated, (**Figure 3A**) whereas Caspase-1 protein levels remained unchanged in OA chondrocytes (**Figure 3B**). CASP1 gene expression in non-OA-chondrocytes was upregulated under both TNF-α concentrations (**Figure 3C**) with the same for protein expression (**Figure 3D**).

In the micromass pellets, 1 ng/ml TNF-α induced both, protein and gene expression of caspase-1 in OA chondrocytes (**Figures 3E, F**). In the non-OA micromass pellets, only protein expression of Caspase-1 was induced (**Figure 3H**), whereas TNF-α stimulation (both conc., 1 ng/mL and 5 ng/mL) did not change gene expression (**Figure 3G**).

Therefore, 1 ng/mL TNF-α was selected as the optimal condition for establishing a chronic low-grade inflammatory model in all subsequent experiments.

### 3.3 Effects of VX-765 on Caspase-1 activity and metabolism of chondrocytes

To assess the effects of the Caspase-1 inhibitor VX-765 on chondrocyte behavior and metabolism, we performed functional assays exclusively in 2D monolayer cultures of OA- and non-OA cells, as these assays have not been validated for micromass pellets.

In OA chondrocytes, VX-765 inhibited Caspase-1 activity without affecting metabolic activity (cell viability), proliferation and senescence but selectively enhanced migration under inflammatory condition.

**Figure 4:**
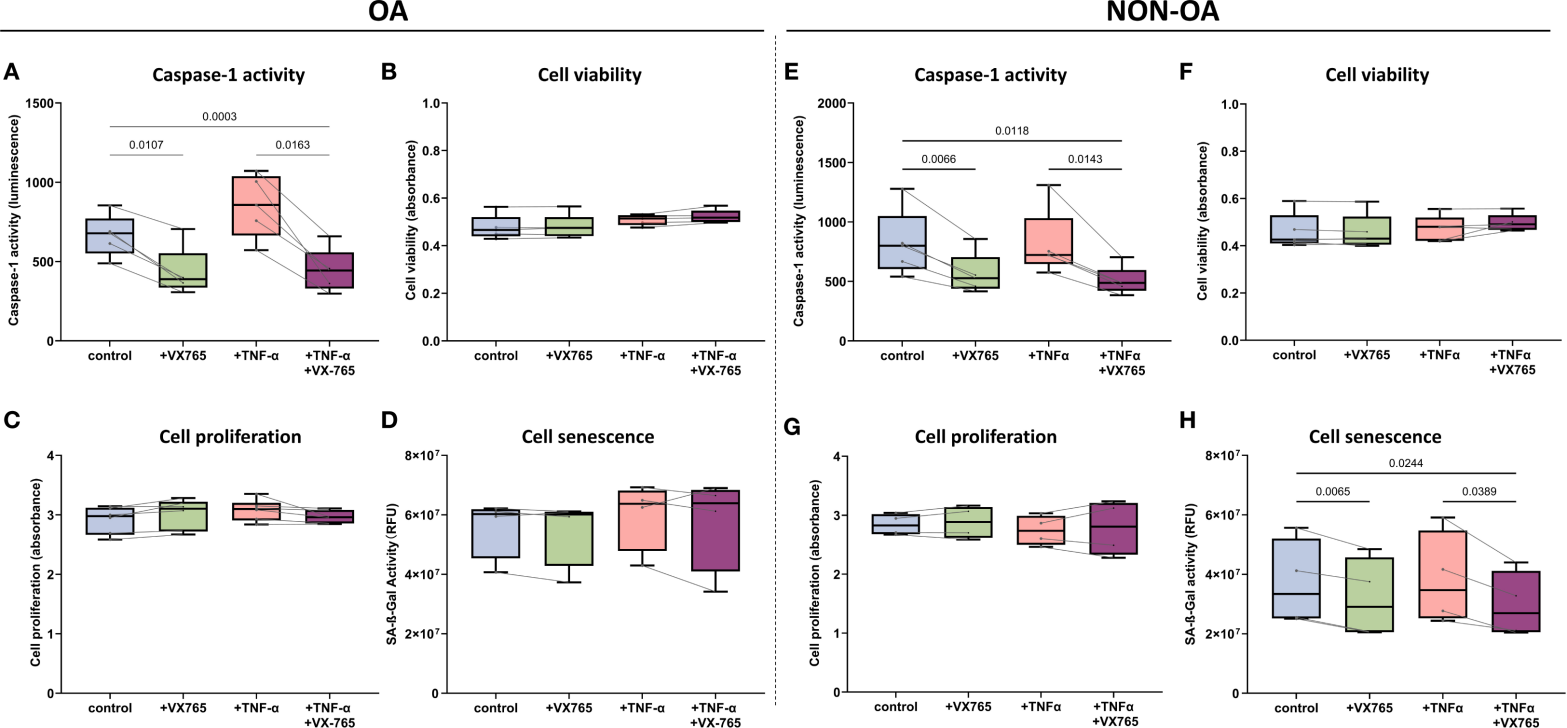
Functional effects of the Caspase-1 inhibitor VX-765 in OA- and non-OA chondrocytes in the presence and absence of TNF-α stimulation. **(A–D)** OA chondrocytes: Following a 24 hour treatment with DMSO only (control), VX-765 (100μM), TNF-α (1 ng/mL), or their combination, OA chondrocytes were assessed for **(A)** Caspase-1 enzymatic activity, **(B)** cell viability, **(C)** cell proliferation, and **(D)** cellular senescence. **(E–H)** Non-OA chondrocytes: Following a 24 hour treatment with DMSO only (control), VX-765 (100μM), TNF-α (1 ng/mL), or their combination, non-OA chondrocytes were assessed for **(E)** caspase-1 enzymatic activity, **(F)** cell viability, **(G)** cell proliferation, and **(H)** cellular senescence. Box plots display median (horizontal line), interquartile range (box), and individual data points. Statistical analysis was performed using RM two-way ANOVA with the Geisser-Greenhouse correction followed by Šídák’s multiple comparisons test (N = 4-6). P-values < 0.05 were considered significant. P-values are indicated where significant differences were observed. To compare the heterogeneity of VX-765 responses, we calculated the coefficient of variation (CV) of the VX-765 response (see **Supplementary Table S5**).

**Figure 5:**
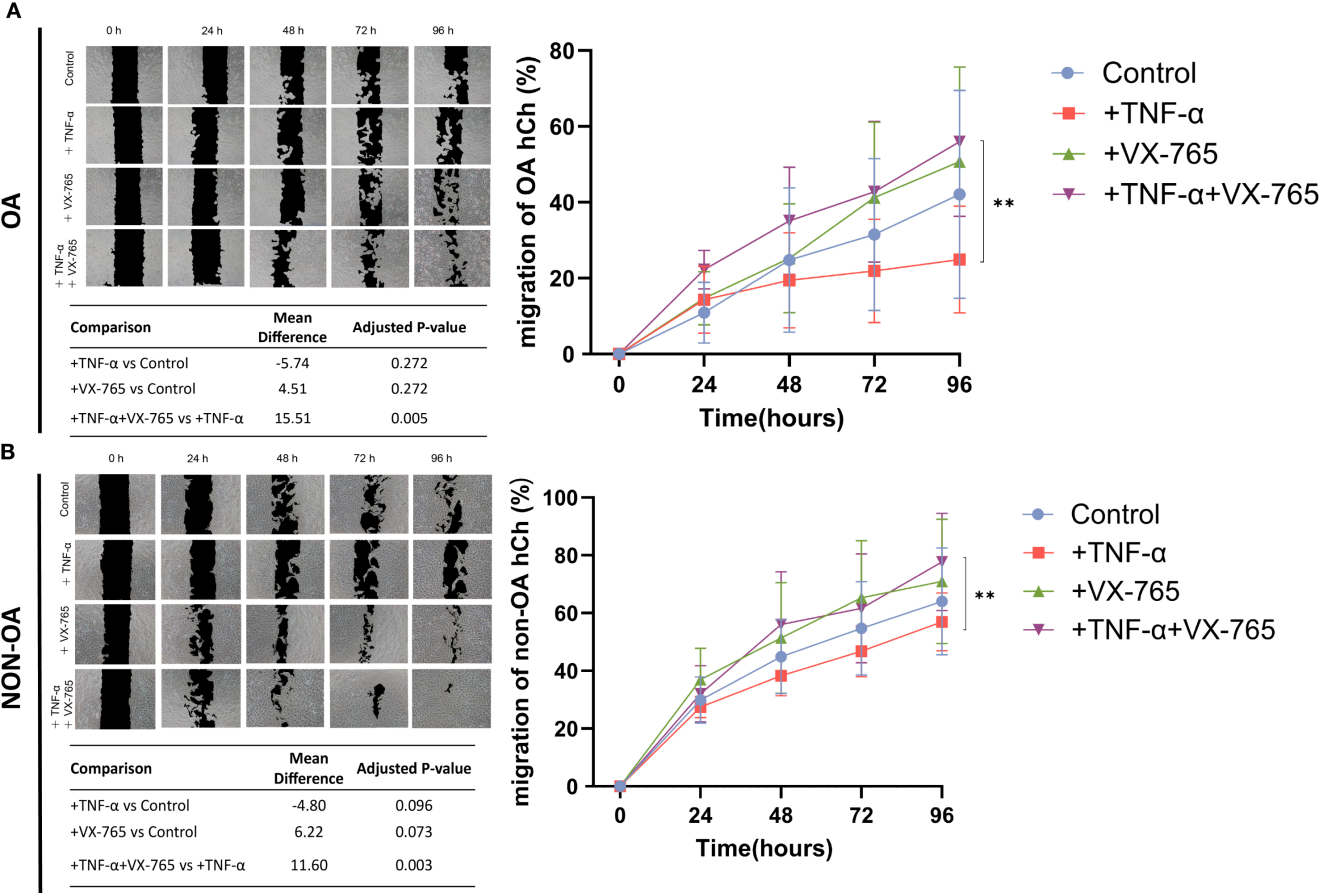
Effects of Caspase-1 inhibitor VX-765 on the migration of OA- and non-OA chondrocytes in the presence and absence of TNF-α. **(A)** Representative images from a scratch (wound-healing) assay illustrate the migration of OA chondrocytes (OA hCh) (over time (0, 24, 48, 72, and 96 hours) under four treatment conditions including reduced serum (1% FCS): control (DMSO only), +TNF-α (1 ng/mL), +VX-765, and +TNF-α combined with VX-765. Black regions denote the initial wound area, while lighter areas indicate areas covered by chondrocytes. The quantitative line graph (right side) shows the percentage of wound closure (mean ± SD) in OA-hCh, with a summary table listing mean differences and adjusted p-values from pairwise comparisons. **(B)** A similar experimental setup was applied to non-OA chondrocytes (non-OA hCh), and representative images were captured at the same time points to assess migration (left panel). The corresponding line graph (right side) displays the wound closure percentage for each Non-OA-hCh treatment group, with the summary table providing statistical comparisons. Statistical analysis was performed using one-way ANOVA followed by Holm-Sidak multiple comparison tests (N = 4). p < 0.05 was considered significant **p<0.01. To reflect the heterogeneity of VX-765 responses, we calculated the coefficient of variation (CV) of the VX-765 response (see **Supplementary Table S5**).

At 100 μM, VX-765 reduced Caspase-1 activity in OA chondrocytes by approximately 50% under both basal (no TNF-α) and TNF-α–stimulated condition (**Figure 4A**). Despite this inhibition, metabolic activity (cell viability), proliferation, and senescence remained unchanged under either basal conditions or TNF-α stimulation (**Figures 4B–D**). Importantly, VX-765 significantly induced OA chondrocyte migration under TNF-α–stimulated conditions (**Figure 5A**), whereas no significant effect was observed under basal condition (no TNF-α).

In non-OA chondrocytes, VX-765 inhibited Caspase-1 activity and reduced senescence without affecting metabolic activity (cell viability) and proliferation, while enhancing migration specifically under inflammatory condition (TNF-α–stimulated condition).

VX-765 reduced Caspase-1 activity by approximately 30% in non-OA chondrocytes (**Figure 4E**). Despite this inhibition, cell viability and proliferation were unaffected under both basal (no TNF-α) and TNF-α–stimulated conditions (**Figures 4F–G**). Importantly, VX-765 consistently reduced senescence, as evidenced by decreased SA-β-Gal activity under both basal (no TNF-α) and TNF-α–stimulated conditions (**Figure 4H**). For migration, VX-765 significantly enhanced non-OA chondrocyte migration under TNF-α–stimulated conditions (**Figure 5B**), whereas no significant effect was observed under basal condition (no TNF-α).

Although 1 ng/mL TNF-α induced only mild, non-significant changes in senescence and migration (**Figures 4**, **5**), VX-765 treatment markedly enhanced migration and reduced senescence, particularly in non-OA chondrocytes.

Taken together, these findings emphasize that VX-765 modulates cellular behavior specifically under inflammatory conditions, underscoring its therapeutic relevance for OA-associated chronic inflammation while preserving normal cellular functions.

### 3.4 Spatial distribution of Caspase-1 in micromass pellets

To investigate the spatial expression of pro- and active Caspase-1 in chondrocyte 3D cultures, and to determine how VX-765 modulates this distribution under inflammatory conditions, we performed histological analysis using the micromass pellet culture system.

In untreated pellets, both pro-Caspase-1 and the cleaved P20 fragment (representing active Caspase-1) were predominantly localized to the peripheral regions (**Supplementary Figure 3**).

Under TNF-α–stimulated condition, VX-765 reduced pellet size, increased pellet density, and shifted Caspase-1 expression from the periphery toward the central regions. This redistribution was consistently observed for both pro-Caspase-1 and active Caspase-1 (P20) (**Supplementary Figure 3**).

These findings indicate that Caspase-1 displays region-specific expression in cartilage tissue, while VX-765 treatment modifies its spatial distribution and is associated with altered tissue morphology.

### 3.5 Effect of Caspase-1 inhibition on MMP gene and protein expression

Given the spatial distribution of Caspase-1 expression and its modulation by VX-765, we hypothesized that Caspase-1 may be linked to ECM metabolism. Since tissue matrix degradation is largely mediated by matrix metalloproteinases (MMPs), with MMP-13 being the key enzyme in cartilage breakdown during OA, we next analyzed the association between Caspase-1 and MMP expression and evaluated the effects of VX-765 on MMP secretion.

**Figure 6:**
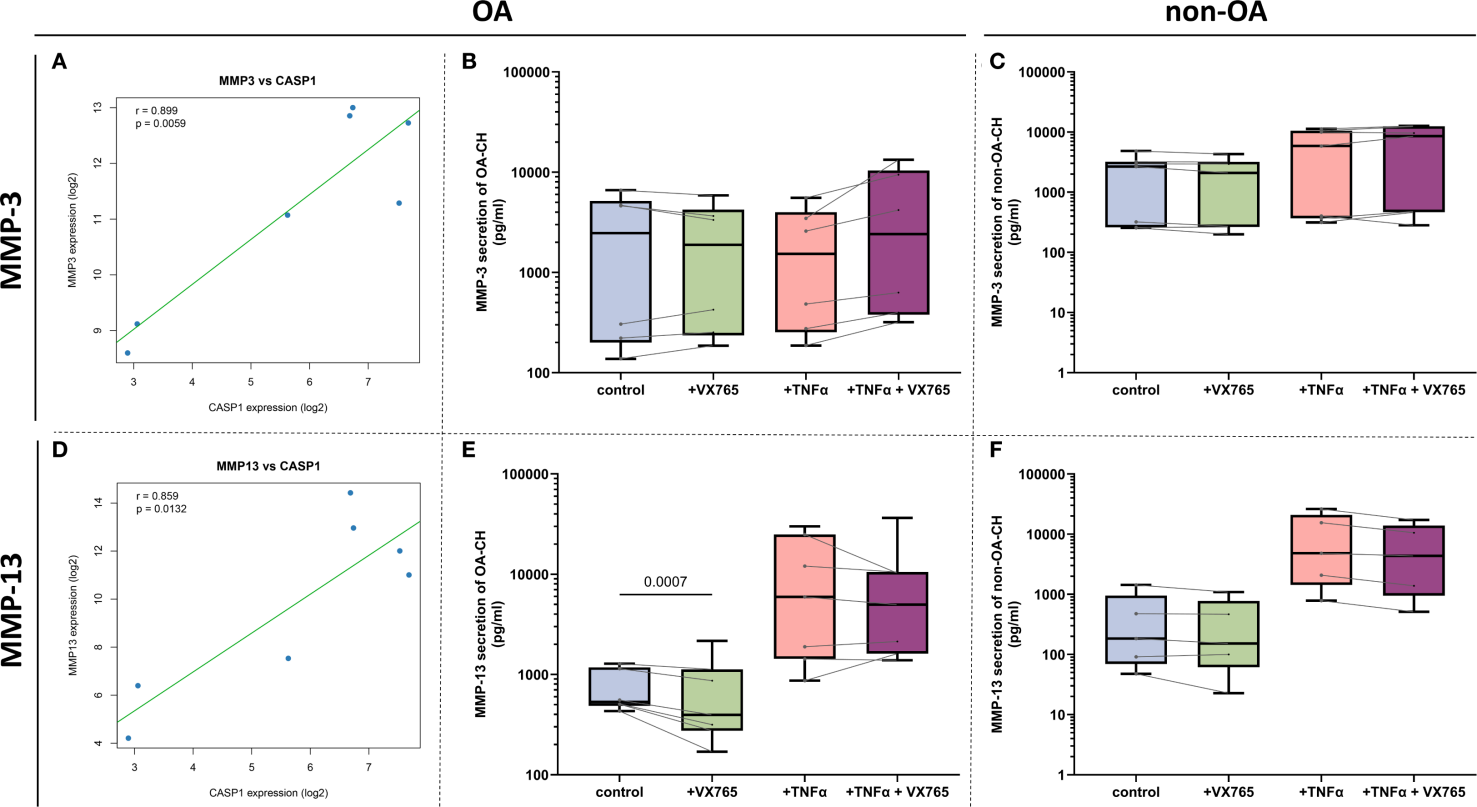
Correlations between CASP1 and MMP gene expression and VX-765 effects on MMP- secretion. **(A, D)** Pearson correlation analysis using bulk mRNA sequencing data (GSE168505) demonstrating significant positive associations between CASP1 gene expression and MMP13 (*r* = 0.859, *P* = 0.0132) and MMP3 (*r* = 0.899, *P* = 0.0059) expression. Data are presented as scatter plots with each point representing one OA- or non-OA patient sample. The solid green line indicates the best-fit linear regression; blue dots denote the 95% confidence interval. Statistical significance was determined at α = 0.05. **(B, C, E, F)** Effects of Caspase-1 inhibitor VX-765 and TNF-α on MMP-13 and MMP-3 protein secretion in pg/mL concentrations from OA- and non-OA chondrocyte supernatants (N = 6 for OA- and non-OA chondrocytes). Statistical significance was assessed using two-way ANOVA with Geisser-Greenhouse correction followed by Šídák’s multiple comparisons test. Data were organized with time points as matched repeated measures. Adjusted P-values were reported, and differences with P < 0.05 were considered statistically significant.

Bulk RNA-seq analysis of seven human cartilage samples (4 OA, 3 non-OA) suggested potential positive correlations between CASP1 and both MMP3 (r = 0.899, p = 0.0059) and MMP13 (r = 0.859, p = 0.0132) (**Figures 6A, D**).

Luminex Multiplex assays of culture supernatants showed that VX-765 significantly suppressed MMP-13 secretion only in OA chondrocytes under basal condition (no TNF-α), with inconsistent effects under TNF-α stimulation (**Figures 6E, F**). In contrast, no significant effects were observed on MMP-3 (**Figures 6B, C**), MMP-2 (**Supplementary Figure 4**), or other MMPs (MMP-1, -7, -9; data not shown) under any condition.

Together, these results suggest that Caspase-1 is functionally linked to MMP-13 regulation in OA chondrocytes, and that VX-765 may modulate cartilage matrix degradation primarily through effects on MMP-13 rather than other MMPs.

### 3.6 Multi-omics analysis reveals Caspase-1 as a central regulator in OA

#### 3.6.1 Transcriptomic analysis shows Caspase-1 classical and non-classical pathway involvement in OA

**Figure 7:**
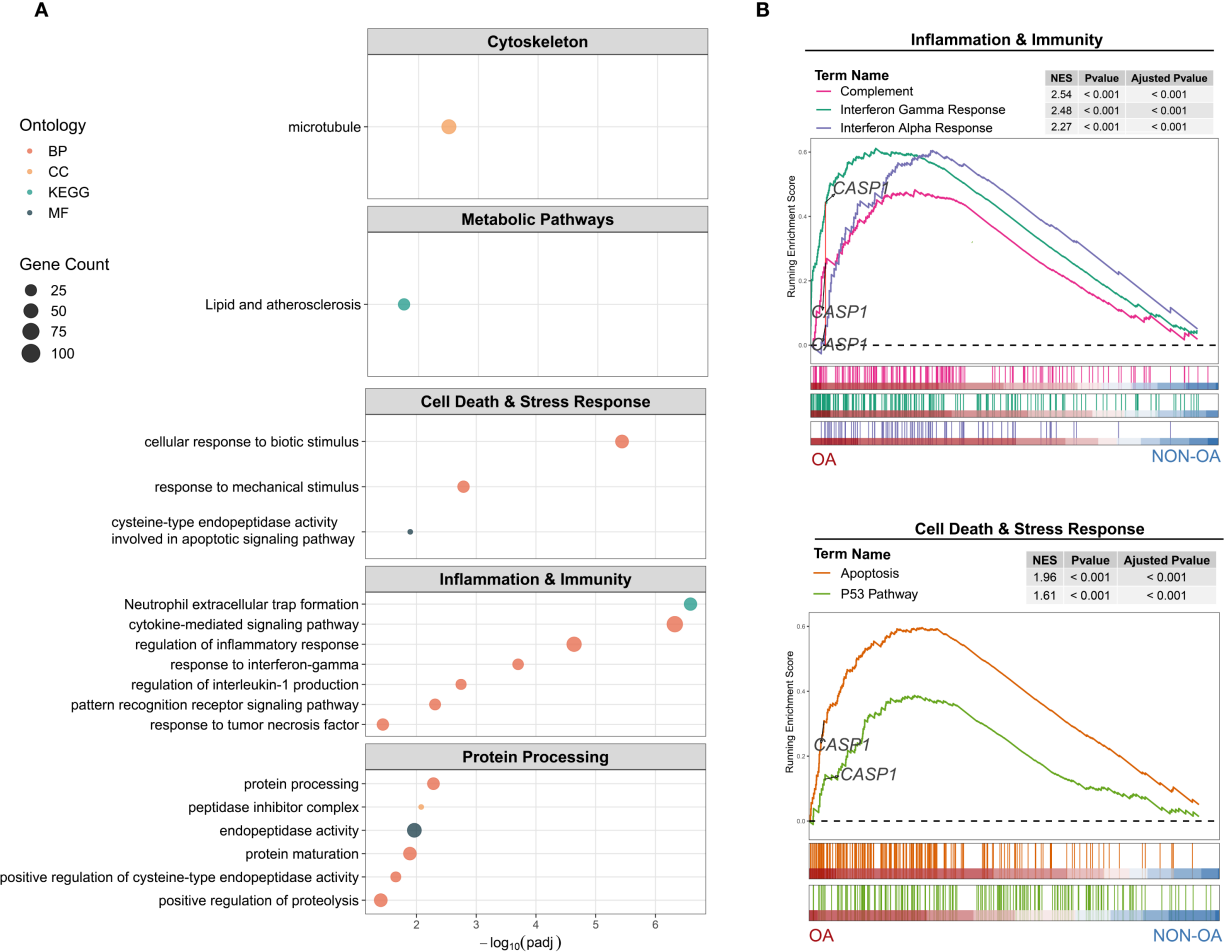
Functional enrichment of CASP1–associated gene expression differences in OA- versus non-OA chondrocytes. **(A)** Over-representation analysis (ORA): Dot plots show the top enriched terms across five functional modules: Cytoskeleton (Cellular Components), Metabolic Pathways (Lipid), Cell Death & Stress Response (Biological Process and Molecular Function), Inflammation & Immunity (Biological Process), and Protein Processing (activity, processing, maturation). Dot size represents the number of differential expressed genes (DEGs) in each term; dot color indicates ontology category. Terms are ordered by –log_10_ (adjusted P-value). **(B)** Gene set enrichment analysis (GSEA): Running enrichment score plots for (top) Inflammation & Immunity Hallmark sets—Complement (red), Interferon Gamma Response (green), and Interferon Alpha Response (violet)—and (bottom) Cell Death & Stress Response Hallmark sets—Apoptosis (orange) and P53 Pathway (olive). Vertical bars beneath each curve mark positions of gene set members in the rank-ordered list from OA (left, red) to non-OA (right, blue). Inset tables list normalized enrichment score (NES), nominal P-value and adjusted P-value for each gene set. The dashed horizontal line indicates ES = 0; CASP1’s location on each plot is labeled.

To explore the pathological role of Caspase-1 in OA, we first performed pathway enrichment analysis comparing OA- and non-OA chondrocytes. Caspase-1 was enriched not only in inflammation-related pathways, such as IL-1 and cytokine signaling, but also in processes including cytoskeletal organization, protein processing, and lipid metabolism (**Figures 7A, B**). Moreover, protein–protein interaction analysis revealed that CASP1 acts as a hub gene within most of these networks (**Supplementary Figure 5**).

These findings indicate that Caspase-1 serves as a central regulator in OA, driving inflammatory signaling and, directly or indirectly (e.g., through inflammation), contributing to broader pathological processes.

#### 3.6.2 Proteomic profiling and molecular docking reveal VX-765-induced protein network remodeling in OA chondrocytes

To validate transcriptomic findings and explore how VX-765 reshapes molecular regulatory networks in OA chondrocytes, we performed LC–MS/MS-based quantitative proteomic profiling analysis and molecular docking analysis, with a particular focus on potential interactions between Caspase-1 and downstream effectors.

**Figure 8:**
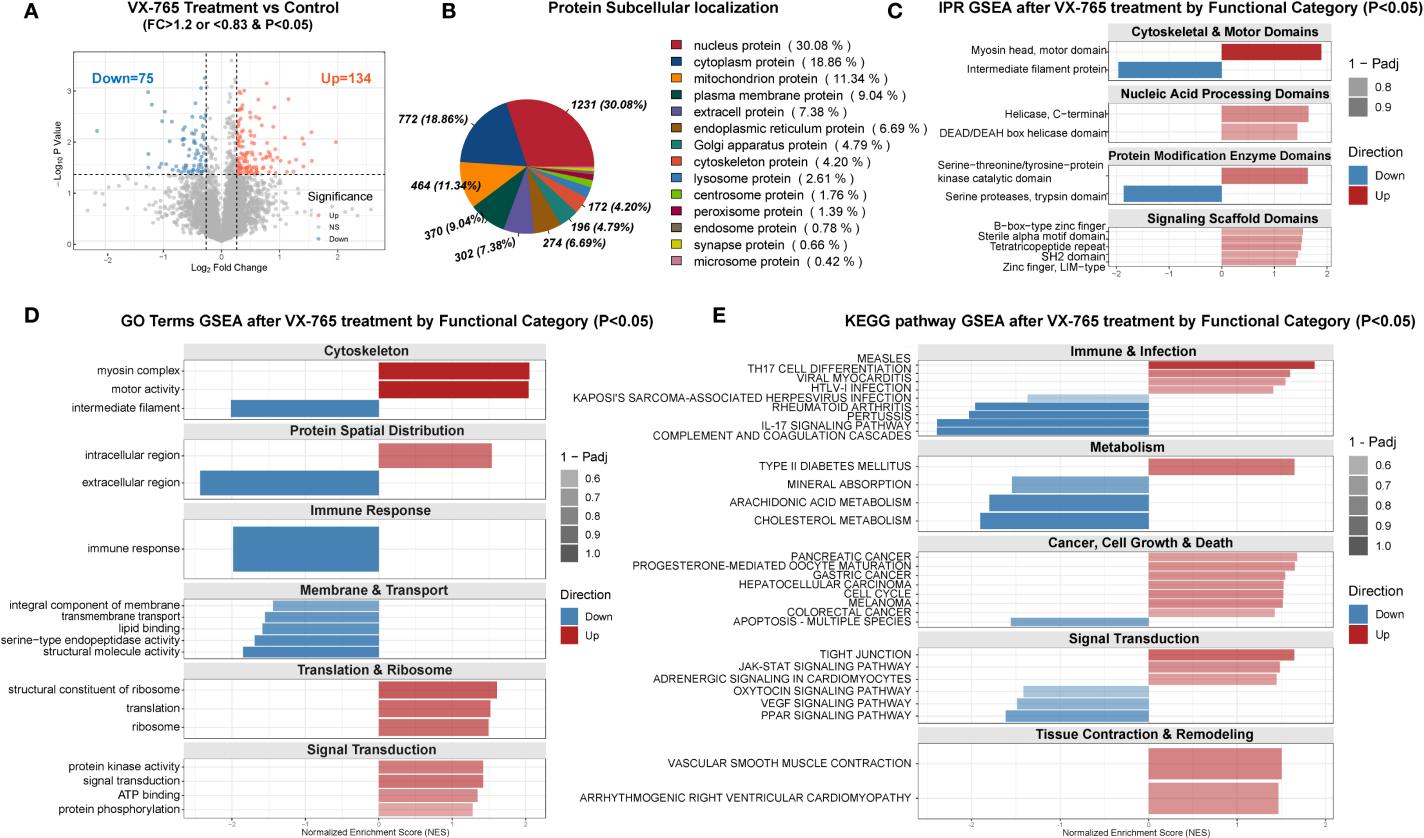
Proteomic profiling of VX-765–treated OA chondrocytes versus controls. **(A)** Volcano plot of differentially expressed proteins in VX-765–treated chondrocytes versus control cells (no treatment). Proteins with fold change (FC) >1.2 and P < 0.05 are shown in red (upregulated, n = 134), and those with FC <0.83 and P < 0.05 in blue (downregulated, n = 75). Gray dots denote non-significantly changed proteins. Dashed lines indicate FC and P-value cutoffs. **(B)** Subcellular localization of all differentially expressed proteins. The pie chart displays the proportion and number (%) of proteins assigned to each compartment (nucleus, cytoplasm, mitochondria, plasma membrane, extracellular/ER, Golgi, cytoskeleton, and other). **(C)** InterPro (IPR) domain–level GSEA of differentially expressed proteins, stratified by functional categories. Bars represent normalized enrichment scores (NES) for domains significantly enriched (P < 0.05); red bars, domains enriched among upregulated proteins; blue bars, domains enriched among downregulated proteins. Bar transparency reflects 1 – Padj. **(D)** Gene ontology (GO) term GSEA by functional module. For each category (Cytoskeleton; Protein Spatial Distribution; Immune Response; Membrane & Transport; Translation & Ribosome; Signal Transduction), bars show NES for significantly enriched GO terms (P < 0.05), with red indicating upregulated and blue downregulated protein sets. **(E)** KEGG pathway GSEA by functional category. Bars display NES for pathways in Immune & Infection; Metabolism; Cancer, Cell Growth & Death; Signal Transduction; and Tissue Contraction & Remodeling (P < 0.05). Color and transparency conventions as in **(C, D)**. The complete proteomic differential expression and enrichment analysis results are provided in **Supplementary Table S6**. In all GSEA panels, NES > 0 indicates enrichment among upregulated proteins, NES < 0 among downregulated proteins. Panels **(C–E)** include only terms with P < 0.05.

Proteomic analysis of VX-765-treated OA chondrocytes identified 5,606 proteins (see **Supplementary Table S2**), with 154 upregulated and 75 downregulated proteins (FC > 1.2 or FC < 0.83, P < 0.05) (**Figure 8A**).

Subcellular localization analysis showed differentially expressed proteins distributed across multiple compartments, with the highest proportions in the nucleus (30.1%) and cytoplasm (18.8%) (**Figure 8B**).

Enrichment analysis further revealed that the differentially regulated proteins were involved not only in inflammatory pathways (e.g., NOD/TNF signaling and immune responses) but also in cell-death regulation (apoptosis and TP53 signaling), as well as broader processes including protein homeostasis, cytoskeletal organization, vesicle trafficking, and stress- and metabolism-related pathways (**Supplementary Figure 6**).

PPI analysis of the 209 proteins identified five main functional modules (mitochondrial translation/protein synthesis, cellular stress response, viral defense, nucleocytoplasmic transport, and energy/nucleotide metabolism) and highlighted 12 hub protein: SMAD2, ABL1, VCAM1, WTAP, POLR2E, THOC2, BIRC2, FAU, TRIP12, MRPL16, MRPS11, and AFG3L2 (**Supplementary Figure 7**).

**Figure 9:**
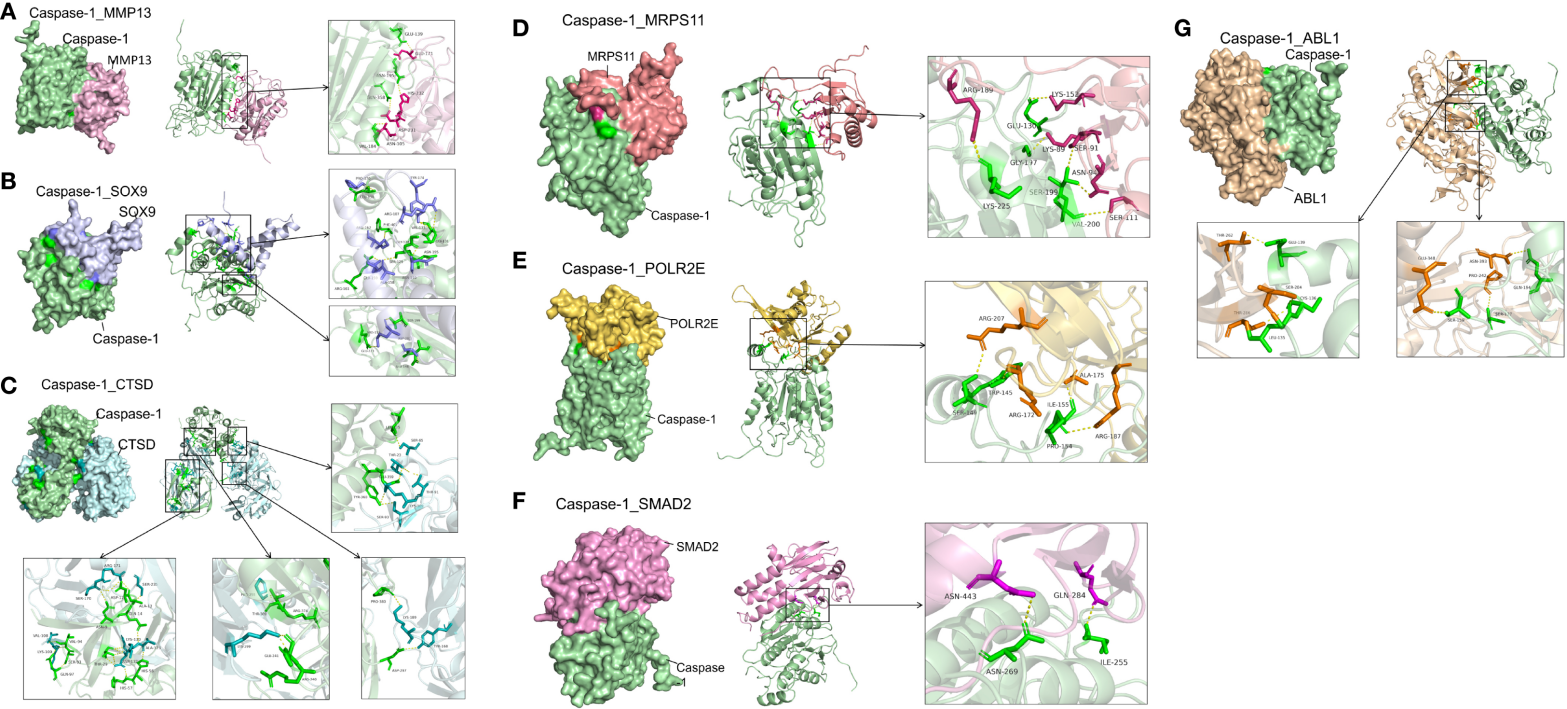
High‐affinity interactions between Caspase-1 and -related proteins revealed by molecular docking. **(A)** Caspase-1 and Matrix Metalloprotease (MMP)-13. Docking of Caspase-1 (green) with MMP-13 (magenta) yielded ΔG = –10.0 kcal/mol. Key interface residues include Glu139 and Asn105 on Caspase-1, and His232 and Asn195 on MMP-13. **(B)** Caspase-1 and SRY-Box-Transkriptionsfaktor (SOX) 9. Caspase-1 (green) in complex with SOX9 (lavender) showed ΔG = –8.0 kcal/mol. Interface residues Arg179 and Cys285 (Caspase-1) and Asp64 and Glu67 (SOX9) are highlighted. **(C)** Caspase-1 and Cathepsin D (CTSD). The Caspase-1 (green)–CTSD (cyan) interaction had ΔG = –6.0 kcal/mol. Contact residues Val184 and His237 (Caspase-1) and Tyr66 and Asp295 (CTSD) are emphasized in the inset. **(D)** Caspase-1 and Mitochondrial Ribosomal Protein S11 (MRPS11). Docking predicts ΔG = –8.1 kcal/mol between caspase-1 (green) and MRPS11 (salmon); Arg189 and Lys225 on Caspase-1 and Lys152 and Ser111 on MRPS11 form the binding interface. **(E)** Caspase-1 and RNA Polymerase II Subunit E (POLR2E). The Caspase-1 (green)–POLR2E (gold) complex exhibits ΔG = –12.5 kcal/mol, with interactions mediated by Ser146, Trp145, and Arg172 (Caspase-1) and Arg207, Ile155, and Ala175 (POLR2E). **(F)** Caspase-1 and Mothers against decapentaplegic homolog 2 (SMAD2). Caspase-1 (green) docking to SMAD2 (pink) shows ΔG = –8.3 kcal/mol. Interface residues include Asn269 and Ile255 on Caspase-1 and Asn443 and Gln284 on SMAD2. **(G)** Caspase-1 and Proto-Onkogen 1, non-rezeptor Tyrosinkinase (ABL1). The Caspase-1 (green)–ABL1 (tan) interaction has ΔG = –11.5 kcal/mol, with key contacts at Leu126, Ser264, and Thr267 (ABL1) and Pro242, Gln194, and Ile120 (Caspase-1). Hydrogen bonds are shown as yellow dashed lines.

To explore potential Caspase-1 interactions, we performed in silico molecular docking with representative molecule and hub proteins. The aim was to determine whether Caspase-1 can directly regulate pathway molecules beyond inflammation, rather than acting only indirectly through downstream inflammatory mediators. Molecular docking revealed strong binding affinities between Caspase-1 and MMP13, CTSD, ABL1, MRPS11, POLR21, SMAD2, and SOX9 (ΔG < –6 kcal/mol; **Figures 9A–G**), suggesting that Caspase-1 may fine-tune OA pathogenesis through direct protein–protein interactions.

These results suggest that VX-765 reprograms key regulatory networks in OA chondrocytes by modulating functional protein modules and hub proteins that integrate inflammatory, metabolic, and structural pathways.

#### 3.6.3 GSEA and cross-omics analyses confirm VX-765 therapeutic potential

To comprehensively evaluate the functional consequences and therapeutic potential of VX-765, we integrated proteomic and transcriptomic analyses. Overall, VX-765 reprograms OA chondrocytes, broadly suppressing disease-associated pathways and promoting a shift toward a healthier phenotype, with selective exceptions.

Proteomic GSEA of 5,606 quantified proteins revealed coordinated rewiring of cellular functions (**Figures 8C–E**). Protein domain enrichment indicated enhanced cytoskeletal motors and kinase/scaffold modules alongside reduced intermediate filaments, suggesting remodeling of structural and interaction networks (**Figure 8C**). Gene Ontology and KEGG enrichment further confirmed increased intracellular activity and biosynthetic processes, suppression of classical pro-inflammatory pathways, downregulation of lipid metabolism and PPAR/VEGF signaling, and activation of JAK–STAT pathways, among others (**Figures 8D, E**).

**Figure 10:**
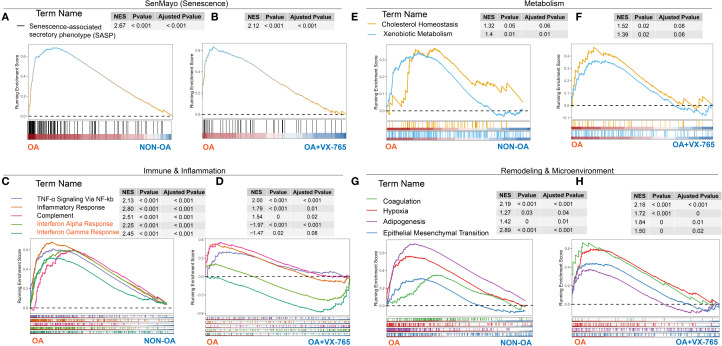
Cross-omics gene set enrichment analysis of OA- versus non-OA transcriptomes and VX-765–treated proteomes. GSEA running‐enrichment plots for four functional modules—senescence **(A, B)**, immunity/inflammation **(C, D)**, metabolism **(E, F)** and remodeling/microenvironment **(G, H)**—with transcriptomic OA- versus Non-OA results in panels **(A, C, E, G)** and proteomic VX-765 treated OA chondrocytes versus control results in panels **(B, D, F, H)**. In each plot, the x-axis represents genes (transcriptome) or proteins (proteome) ranked from OA (left) to non-OA or VX-765-treated (right), the y-axis shows the running enrichment score (ES) (dashed line = 0), colored curves denote individual gene sets, tick marks indicate gene positions, and inset tables list NES, P-value and FDR (Adjusted P-value).

Transcriptomic analysis revealed enrichment of OA-associated signatures, including SASP (**Figure 10A**), TNF-α/NF-κB, interferon responses, complement activation (**Figure 10C**), altered cholesterol/xenobiotic metabolism (**Figure 10E**), and remodeling modules such as adipogenesis, hypoxia, and EMT (**Figure 10G**). At the proteomic level, VX-765 markedly suppressed these OA-associated pathways, effectively reprogramming the chondrocyte profile toward a non-OA–like state (**Figures 10B, D, F, H**). Notably, however, VX-765 induced a pronounced activation of interferon-α/γ signaling compared with OA controls (**Figures 10C, D**).

Together, these findings indicate that VX-765 broadly reprograms OA-associated pathways toward a healthier state, underscoring its therapeutic potential, while the induction of interferon signaling represents a selective exception.

### 3.7 Population-based analysis supports Caspase-1 as a therapeutic target and reveals genetic heterogeneity

Given the substantial inter-individual variability observed in chondrocyte experiments, we hypothesized that genetic variation in the CASP1 locus may differentially influence OA susceptibility. To test this, we performed Mendelian randomization analysis using expression quantitative trait loci (eQTL) for CASP1 and its regulatory genes (CARD17, CARD18, and CARD8) in peripheral blood, selecting variants within ±100 kb of each gene. Large-scale OA GWAS datasets were used as outcomes.

**Figure 11:**
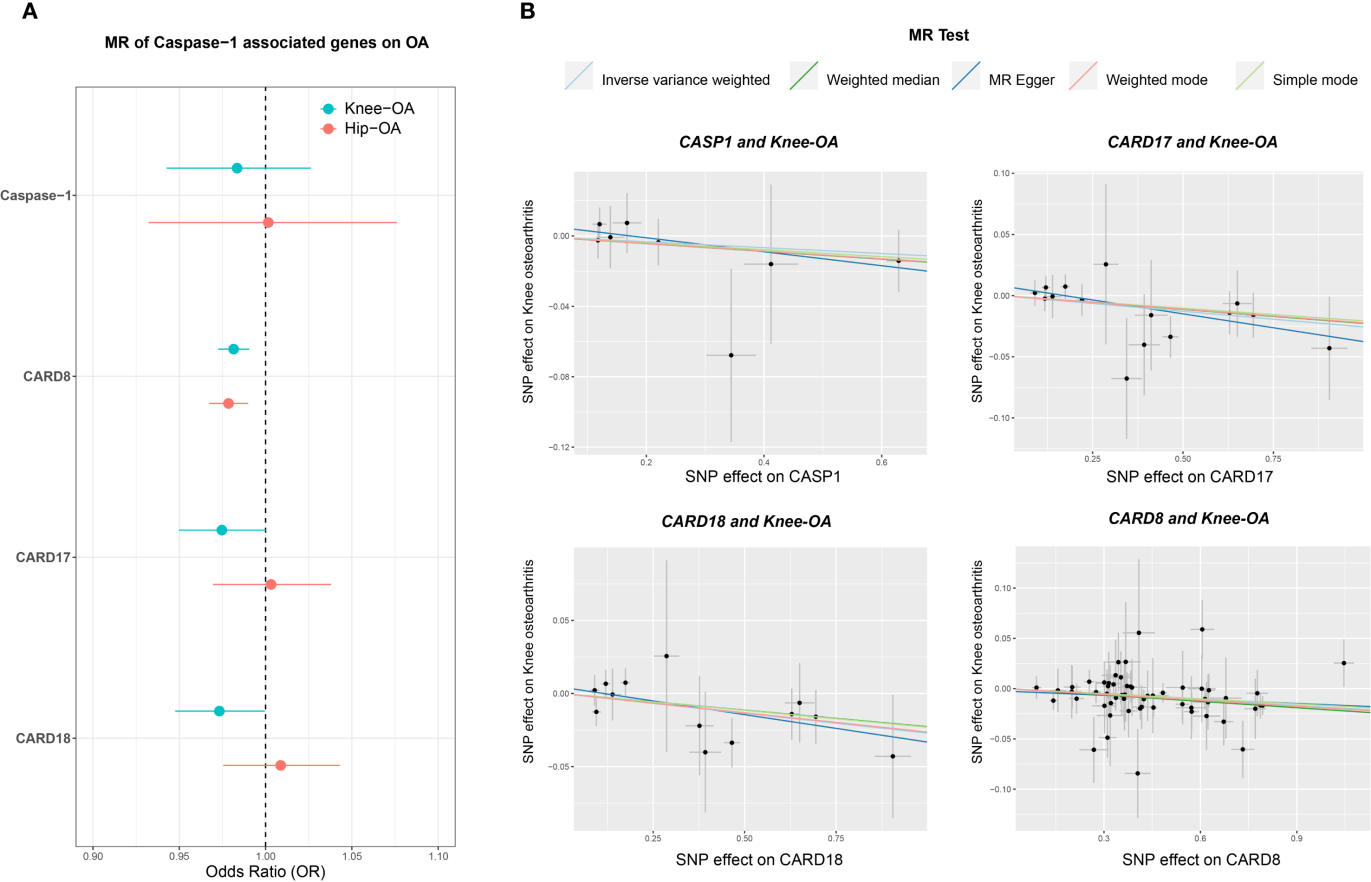
Mendelian randomization (MR) analysis of CASP1-associated genes in knee and hip OA. **(A)** Forest plot displaying odds ratios (ORs) and 95% confidence intervals (CIs) for CASP1, CARD8, CARD17, and CARD18, comparing their effects on knee OA (teal circles) and hip OA (orange circles) risks. The vertical dashed line at OR = 1 indicates no effect on OA risk. **(B)** Scatter plots of MR estimates (e.g., IVW, MR-Egger, Weighted Median) showing the genetic instrument-exposure relationship on the x-axis (SNP–gene expression) and the instrument–outcome relationship on the y-axis (SNP–OA). Each line represents a different MR method, with its slope indicating the estimated causal effect. The analyses were performed under standard MR assumptions, and statistical significance was evaluated using multiple MR methods. These results suggest gene-specific patterns of association for CASP1 and its inhibitory proteins (CARD8, CARD17, CARD18) with knee and hip OA.

MR analysis revealed that genetically predicted higher expression of CARD17, CARD18, and CARD8 was significantly associated with decreased risk of knee OA, with consistent trends in hip OA (**Figure 11A**). In contrast, associations for CASP1 expression showed greater heterogeneity and did not reach statistical significance overall. Nonetheless, predicted higher CASP1 expression was negatively associated with OA risk (**Figure 11B**), suggesting that certain CASP1 variants may confer a protective effect on cartilage homeostasis.

To address potential confounding from linkage with neighboring genes (CARD17/18), we repeated MR using only variants located within 100 bp of the CASP1 locus. Results remained consistent, supporting a protective association of CASP1 expression with OA (**Supplementary Figure 8**). Cross-disease comparisons indicated specificity: while CASP1 variants such as rs61751523 and rs536909 showed negative associations with OA, they exhibited positive associations with RA and Crohn’s disease (**Supplementary Figure 9**). Phenome-wide association study (PheWAS) further revealed moderate associations of these variants with lipid metabolism traits (1 × 10^-4^ < P ≤ 0.05) (**Supplementary Figure 10**).

Motif analysis suggested that rs61751523 alters transcription factor binding, with the alternate allele increasing NF-κB binding, reducing FOXP1 and NKX6–1 binding, and modestly enhancing STAT binding (**Supplementary Table S3**).

Data sources, instrument SNPs, and sensitivity analyses are provided in **Supplementary Table S4**.

Collectively, these genetic findings support Caspase-1 and its regulatory network as candidate therapeutic targets in OA, while highlighting genetic heterogeneity that may influence their effects on cartilage homeostasis.

## 4 Discussion

Our findings demonstrate that Caspase-1, also known as interleukin-1β converting enzyme (ICE), and its regulatory molecules, such as components of the NLRP3 inflammasome, are upregulated in OA chondrocytes and are presumably correlated with the expression of genes involved in cellular senescence, inflammation and immunity, oxidative stress, extracellular matrix metabolism, and cell cycle regulation, including expression of MMP13 and MMP3. In both OA- and non-OA chondrocytes, TNF-α stimulation generally enhanced the expression of Caspase-1 at both mRNA and protein levels.

Under conditions of partial inhibition of Caspase-1 activity (a reduction of up to 50%), the specific inhibitor VX-765 consistently reduced cellular senescence in non-OA chondrocytes and promoted migration capacity of both, OA- and non-OA chondrocytes in the presence of TNF-α, while having no significant effect on cell viability or proliferation.

Integrating transcriptomic and proteomic data, we uncovered multiple levels of potential Caspase-1 involvement in OA pathogenesis, encompassing gene expression regulation, protein processing and translational control, vesicular transport and nucleo-cytoplasmic trafficking, cellular senescence, cytoskeletal and junctional remodeling, as well as inflammatory homeostasis and immune-related signaling pathways. VX-765 appears to modulate these critical biological processes, broadly suppressing inflammatory cascades and senescence-associated metabolic pathways while activating cell cycle and repair-related signaling, ultimately promoting a shift toward metabolic homeostasis in chondrocytes. Mendelian randomization analysis in human populations supports a causal relationship between increased genetically predicted levels of Caspase-1 inhibitory molecules (CARD17, CARD18, and CARD8) and reduced risk of knee OA.

### 4.1 Caspase-1 in OA chondrocytes and its pathological significance

Caspase-1 is well-recognized for its essential role in classical inflammatory responses and host defense against pathogens. Structurally, Caspase-1 comprises an N-terminal caspase recruitment domain (CARD) and protease domains containing the catalytic cysteine residues (P20 and P10) (28). Many studies have confirmed that inhibition or genetic deletion of Caspase-1 can significantly alleviate inflammation in various disease models, including neurodegenerative and cardiovascular conditions (21, 22). Although the development of Caspase-1-targeting drugs remains challenging, most small-molecule inhibitors developed to date target its catalytic site (19). These inhibitors typically exhibit reversibility and regulatory flexibility, allowing a balance between inflammation control and antimicrobial defense, and thus demonstrate promising translational potential. VX-765, a representative specific Caspase-1 inhibitor with favorable pharmacokinetics and low toxicity, has shown therapeutic efficacy in several experimental inflammatory disease models such as Psoriasis and RA (20), however, its long-term *in vivo* usage generated also adverse effects.

Given the shared inflammatory mechanisms among various diseases, VX-765 can be also considered as a potential candidate for OA treatment. However, compared to the autoimmune disorder RA, OA is characterized by low-grade chronic inflammation and a more complex etiology involving mechanical stress, metabolic dysregulation, and other non-inflammatory factors (9). These features contribute to the uncertainty surrounding the efficacy of VX-765 as therapeutic agent in OA. Moreover, emerging studies have revealed non-canonical functions of Caspase-1, such as its involvement in unconventional protein secretion and regulation of cartilage nodule formation, further complicating prediction of its role in OA pathophysiology (15, 16).

In this study, we initially analyzed publicly available chondrocyte transcriptomic datasets (GSE168505). In silico, we observed significant upregulation of Caspase-1, as well as inflammasome-related factors such as NLRP3 and several pro-inflammatory cytokines in OA chondrocytes compared to non-OA chondrocytes. These findings support the classification of OA as a “chronic inflammatory disease.” Additionally, we found significant enrichment of genes related to pathways such as cholesterol metabolism and the complement system, confirming that the pathogenesis of OA extends beyond simple inflammation. Further correlation analyses revealed that Caspase-1 expression was closely associated with genes involved in cellular senescence, oxidative stress, extracellular matrix metabolism, and cell cycle regulation. These findings in addition to caspases as potential biomarker for OA prognosis (29) suggest that Caspase-1 may lie at the intersection of multiple regulatory pathways and play a broader role as originally assumed in OA pathogenesis.

Enrichment analysis of differentially expressed genes between OA and non-OA chondrocytes further confirmed the involvement of Caspase-1 in several key signaling pathways, including immune-related pathways such as the NOD-like receptor signaling pathway. These results are consistent with previous findings as Yinzi Xin et al. reported that Caspase-1 inhibition attenuated synovial inflammation in a temporomandibular joint OA model via the NLRP3 inflammasome pathway (23). However, the non-inflammatory functions of Caspase-1 in pathologies as OA remained largely unexplored.

Our data also suggest that Caspase-1 may participate in diverse biological processes including RNA methylation, cytoskeletal remodeling, protein translation, and post-translational modifications. In line with this, under TNF-α stimulation we observed that mRNA and protein levels of Caspase-1 did not always correlate. In some conditions, mRNA levels declined while protein levels increased, implying that Caspase-1 expression also in chondrocytes is strongly influenced by both post-transcriptional and post-translational regulatory mechanisms (30, 31). Our in silico molecular docking analysis predicts high-affinity interactions between Caspase-1 and mitochondrial ribosomal protein (MRP)S11 as well as RNA polymerase subunit POLR21. This further supports the hypothesis that Caspase-1 regulation depends on complex post-transcriptional and post-translational mechanisms. However, as molecular docking provides only predictive insights, future *in vitro* and *in vivo* studies will be essential to validate these interactions and establish their biological significance.

Together, these findings provide evidence for several non-canonical regulatory mechanisms of Caspase-1 in chondrocytes and suggest that its functional regulation extends beyond traditional control of apoptosis.

### 4.2 Effects of Caspase-1 inhibition on OA-associated pathological phenotypes

We next explored how Caspase-1 inhibition affects key OA phenotypes *in vitro*. Using primary human OA and non-OA chondrocytes, we simulated inflammatory stress with TNF-α treatment and applied VX-765 to inhibit Caspase-1 activation. This design allowed us to compare chondrocytes under mild (non-OA + TNF-α) or more severe (OA + TNF-α) inflammatory conditions. Overall, VX-765 treatment conferred beneficial effects on several metabolic parameters, notably reducing cellular senescence and enhancing migratory capacity, while also modulating secretion of matrix-catabolic enzymes.

Overall, it is agreed on that persistent inflammation is a key driver of age-related diseases and that down regulation of aberrant NLRP3 inflammasome activation by inhibition of Caspase-1 activity may reduce the onset or severity of multiple age-related chronic diseases, e.g. OA. Pro-inflammatory cytokines, e.g. TNF-α, show an age-dependent regulation implicating inflammasome mediated caspase-1 activation in the aging process (32). Notably, OA and non-OA chondrocytes exhibited differential responses regarding senescence reduction. In non-OA chondrocytes, VX-765 reduced SA-β-galactosidase activity, whereas in OA chondrocytes VX-765 did not change SA-ß-galactosidase correlated senescence, potentially due to differences in cell autonomous inflammation levels. These results imply VX-765 may be most effective in early or low-inflammation stages of OA, helping to delay inflammatory “aging” of chondrocytes. Supporting this, chronic inflammasome signaling is known to promote a senescence-associated secretory phenotype in cells (33), which VX-765 might counteract in chondrocytes.

Accumulating evidence shows that caspases, especially Caspases-1, -3, -8 and -9, have been linked to cell migration and motility and are crucial for proper cell migration and tissue repair (34). Our data are contrary – VX-765 treatment in the presence of TNF-α induced migration in both OA and non-OA cells - to the recently reported novel function of Caspase-1 in facilitating the migration of hair follicle stem cells into the epidermis through the activation of CARD (35). However, these are mesenchymal precursor cells, which might react differently to stimuli induced by epidermal wounding as fully differentiated chondrocytes, which are surrounded by a dense extracellular matrix. In addition, only in combination with TNF-α, VX-765 treatment did induce migration significantly, without TNF−α we did not detect significant migration induction. We thus hypothesize that Caspase-1 may influence cytoskeletal or matrix-remodeling proteins that govern cell movement. Transcriptomic enrichment showed that Caspase-1 participates in OA-related microtubule remodeling pathways. Proteomic GSEA further revealed a pronounced increase in myosin head/motor domains alongside a decrease in intermediate filament domains after VX-765 treatment, reflecting a shift toward a more dynamic, flexible cytoskeleton.

VX-765 effectively suppressed MMP-13 secretion of OA chondrocytes, with our molecular docking analysis further indicating a potential direct physical interaction between Caspase-1 and MMP-13. This implies that the regulation of MMP-13 secretion by Caspase-1 may involve not only traditional upstream signaling (e.g., by inflammation) but also direct molecular binding of both proteins which consequences need further investigation. In addition, Caspase-1 also showed potential binding interactions with ABL1, MRPS11, POLR21, SMAD2, SOX9 and CTSD, indicating that it may participate directly in chondrogenic differentiation and cartilage homeostasis beyond its known role in inflammation and cell death. The prediction of direct binding includes Caspase-1 and tyrosin kinase ABL1, which phosphorylates actin, and microtubule-associated proteins (e.g., CLASP2, γ-tubulin) to control filament assembly and cell adhesion (36, 37), as well as interactions with MMP-13 and cathepsin D (CTSD), both remodeling the matrix. By inhibiting these non-canonical Caspase-1 interactions, VX-765 likely frees ABL1-driven cytoskeletal remodeling and reduces matrix constraints on migration. Furthermore, senescent cells are known to exhibit impaired regenerative capacity, including reduced migration (38). Thus, VX-765’s dual effects on senescence and migration may have synergistic functional implications.

Interestingly, although these positive effects on reducing senescence and increasing migration, VX-765 did not significantly change cell viability or proliferation. Caspase-1 has its most prominent roles in autophagy and pyroptosis and less in regulation of the cell cycle and thus proliferation contrary to other caspases, e.g. Caspases-3, -7, -8 and -9 (34). Latter are known to play critical roles in proliferation of various cancer cell lines and lymphocytes as T- and B-cells. However, also for these caspases – including Caspase-1 - nothing is reported about possible functions in proliferation and cell cycle regulation of chondrocytes. Notably, our proteomic analysis suggests that VX-765 profoundly remodels mitochondrial architecture: GSEA highlighted upregulation of oxidative phosphorylation and mitochondrial ribosome pathways, and NADH subunits were significantly increased. Molecular docking further predicts a high-affinity interaction between Caspase-1 and chain A Mitochondrial ribosomal protein (MRP) L11, pointing to direct modulation of mitochondrial translation. We also noted substantial donor-to-donor variability: some chondrocyte samples showed modest proliferative or metabolic responses to VX-765, whereas others did not. This heterogeneity may reflect differences in OA subtype, baseline inflammation, or genetic background (e.g., inflammation-associated SNPs). Moreover, our regimen achieved only a max. 50% reduction in Caspase-1 activity suggesting that more sustained or potent inhibition might be required to translate molecular priming into cell expansion. These findings underscore the need to consider patient-specific factors and dosing strategies when evaluating Caspase-1–targeted therapies.

### 4.3 Core pathological pathways mediated by Caspase-1

Taken together, our multi-omics data suggest that in chondrocytes VX-765 not only powerfully downregulates IL-1β/IL-18–driven inflammation, apoptosis and senescence, but also ramps up repair pathways, mitochondrial biogenesis and cytoskeletal remodeling—essentially able to “reprogramming” diseased chondrocytes. Unexpectedly, this reset comes with a strong surge in interferon-α/γ signaling. Although VX-765 severs the NLRP3 axis by blocking Caspase-1 activation (preventing Gasdermin D cleavage, reducing IL-1β/IL-18 release and pyroptosis), whereas DAMPs generated by mechanical stress or mitochondrial DNA damage (e.g. HMGB1, mtDNA, ATP) can still activate STING, which via TBK1 and IRF3/7 drives IFN-α/γ production. That interferon wave may help curb MMP-13 activity (39) and promote cartilage regeneration (40), but chronic STING activation has also been linked to low-grade inflammation and upregulation of MMP-13 and ADAMTS5 through NF-κB and ER stress (41, 42). In other words, it remains unclear whether the VX-765–induced interferon spike is “chondroprotective” or “pro-inflammatory.” At the same time, the non-canonical roles of Caspase-1 in mitochondrial biogenesis, cytoskeletal dynamics, transcriptional regulation and senescence also warrant deeper investigation.

### 4.4 Genetic evidence supporting Caspase-1-related pathways in human OA and population-level heterogeneity

We applied two-sample Mendelian randomization (MR) to investigate how Caspase-1 and its CARD-specific regulators (CARD17, CARD18, CARD8) might influence OA risk via blood eQTLs. Genetically predicted higher expression of CARD17, CARD18, and CARD8 was significantly associated with reduced OA risk, consistent with their ability to bind the CARD domain of pro-Caspase-1 and block inflammasome assembly (18).

In contrast, Caspase-1 itself exhibited more complex, context-dependent effects. We determined specific expression Quantitative Trait Loci (eQTLs) such as rs536909 and rs61751523 as protective in OA but associated with increased risk in RA and Crohn’s disease, highlighting the functional heterogeneity of Caspase-1. PheWAS further linked these SNPs to lipid metabolism traits. Motif analysis of rs61751523 predicted increased NF-κB binding, loss of FOXP1/NKX6–1 sites, and a modest gain in STAT binding—suggesting allele-specific modulation of inflammatory and metabolic transcriptional networks. These findings imply that CASP1 genetic variations may influence OA not only through classical IL-1β/pyroptosis pathways but also via broader effects on lipid homeostasis and gene regulation. Taken together, genetic variation detected by SNP analysis, in the CASP1 gene itself shows greater heterogeneity, with certain SNPs exhibiting protective effects, which may be linked to the involvement of Caspase-1 in cartilage development.

Beyond inflammation, Caspase-1 may also be involved in cartilage biology. In silico performed Molecular docking analyses predicted strong binding between Caspase-1 and the chondrogenic transcription factor SOX9, and prior studies have linked Caspase-1 to CD36-mediated lipid metabolism and cartilage nodule formation—pointing to potential physiological roles in tissue development and repair (16).

### 4.5 Potential biomarkers for VX765 response prediction

In our experiments, we observed considerable inter-individual heterogeneity in response to VX-765, with baseline inflammation emerging as a key determinant. Responses differed between OA- and non-OA chondrocytes and were further modulated by TNF-α stimulation, indicating that an inflammatory context strongly shapes individual sensitivity to VX-765. Therefore, identifying predictive biomarkers of response is essential.

Several potential candidates can be considered. First, molecules involved in NLRP3 inflammasome assembly and activation—such as NF-κB, TNF-α, IL-1β and its receptors, NLRP3, ASC, NEK7, CASP4/5, CARD16/17/18, CARD8, and GSDMD—represent primary candidates, as they are closely linked to Caspase-1 activity and are generally upregulated in OA compared with non-OA conditions. Second, Caspase-1–interacting molecules identified through PPI network analysis, including CXCL chemokines, CCL2/3, and complement factors, may serve as secondary candidates. Third, proteomic data indicate that VX-765 influences stress-response proteins and extracellular matrix remodeling pathways; thus, markers such as COMP, MMP-1, MMP-3, collagen metabolites (CTX-II, C2C, CPII, PIICP), and mitochondrial injury indicators (e.g., circulating cell-free mtDNA) may act as systemic biomarkers of drug response. Finally, genetic variation in CASP1 and its regulators (CARD17/18, CARD8, NLRP3, ASC) offers an additional layer of predictive stratification.

### 4.6 Study limitations and future research directions

Though, our study is among the first to investigate the role of Caspase-1 in human OA using primary human chondrocyte samples alongside population-level analysis, several limitations remain. First, VX-765 is a “pro-drug” that requires enzymatic conversion *in vivo* to exert its inhibitory effect. The discrepancy between *in vivo* and *in vitro* metabolic environments may influence drug activation and efficacy. Additionally, the study used only a single concentration –obtained from literature and the company- without dose–response analysis, limiting our knowledge of its pharmaco-dynamics and safety window. Second, while VX-765 is designed to selectively inhibit Caspase-1, off-target effects on Caspase-4/5 cannot be excluded (20). Therefore, future studies should focus on elucidating the role of Caspase-4/5. Furthermore, since VX-765 targets only the catalytic domain, it may not effectively block upstream inflammasome assembly. Approaches targeting inflammasome assembly or Caspase-1 recruitment may offer complementary therapeutic benefits (43). Third, although we used primary chondrocytes from human OA- and non-OA donors, detailed clinical stratification (e.g., by endotype or comorbidities) was not performed. This limits the generalizability of our findings and the identification of endotype-specific responses. Future studies should involve larger, clinically characterized cohorts to clarify the heterogeneity of Caspase-1’s function in OA. Finally, while enrichment analyses pointed to downstream pathways such as cellular senescence and complement activation, further mechanistic studies are needed to dissect Caspase-1’s non-canonical roles—including potential interactions with Caspase-4/5 and its involvement in mitochondrial function, cytoskeletal remodeling, and transcriptional regulation.

## 5 Conclusion

In summary, this study demonstrates that Caspase-1 expression can be upregulated in non-OA chondrocytes via TNF-α stimulation and is associated with multiple pathological molecular pathways, including cellular senescence, ECM degradation, and metabolic dysregulation. Partial inhibition of its catalytic activity alleviated cellular senescence in non-OA chondrocytes and enhanced chondrocyte migratory capacity without affecting cell viability and proliferation, while also modulating MMP-13 secretion - likely through both inflammation-dependent and independent mechanisms. Integrated transcriptomic and proteomic analyses suggest that OA chondrocytes overexpress Caspase-1 compared to non-OA chondrocytes and that Caspase-1 participates in several non-canonical functional pathways, including RNA modification, ribosome assembly, and cytoskeletal remodeling. Population-level genetic analyses further support a causal relationship between Caspase-1 regulatory factors and KOA risk. Taken together, these data suggest that Caspase-1 inhibition carries strong context dependency. Therapeutic strategies must therefore balance the suppression of pathological Caspase-1 activation with the preservation of its homeostatic functions, particularly during phases of growth (cell expansion) or regeneration (cell migration).

Collectively, our findings position Caspase-1 as a key regulatory node with multidimensional roles in OA pathogenesis. However, therapeutic targeting of Caspase-1 should consider individual variability, inflammatory context, and its physiological roles in development, energy networks and tissue homeostasis. These findings support Caspase-1 as a promising target for personalized OA therapies and encourage the development of selective inflammasome inhibitors in translational orthopedics and rheumatology.

## Data Availability

The datasets used and/or analyzed during the current study are available from the corresponding author on reasonable request.
