# Supplementary material for: Targeting Caspase-1 in osteoarthritis: multi-omics insights into the effects of VX-765 on human chondrocyte function and phenotype
Source: Front Immunol. 2025 Oct 3;16:1677801. doi: 10.3389/fimmu.2025.1677801 (PMC12532135; doi:10.3389/fimmu.2025.1677801)
Supplement: Supplementary file 1 [file DataSheet1.docx]

**Targeting Caspase-1 in Osteoarthritis: Multi-Omics Insights into the Effects of VX-765 on Human Chondrocyte Function and Phenotype**

Jian Mei, Nicole Schäfer, Penghui Wei, Zhiheng Kong, Shushan Li, Patrick Pann, Marianne Ehrnsperger, Brian Johnstone, Eva Matalova, Susanne Grässel#

**Supplementary Figures include:**

**Supplementary Figures 1-10**


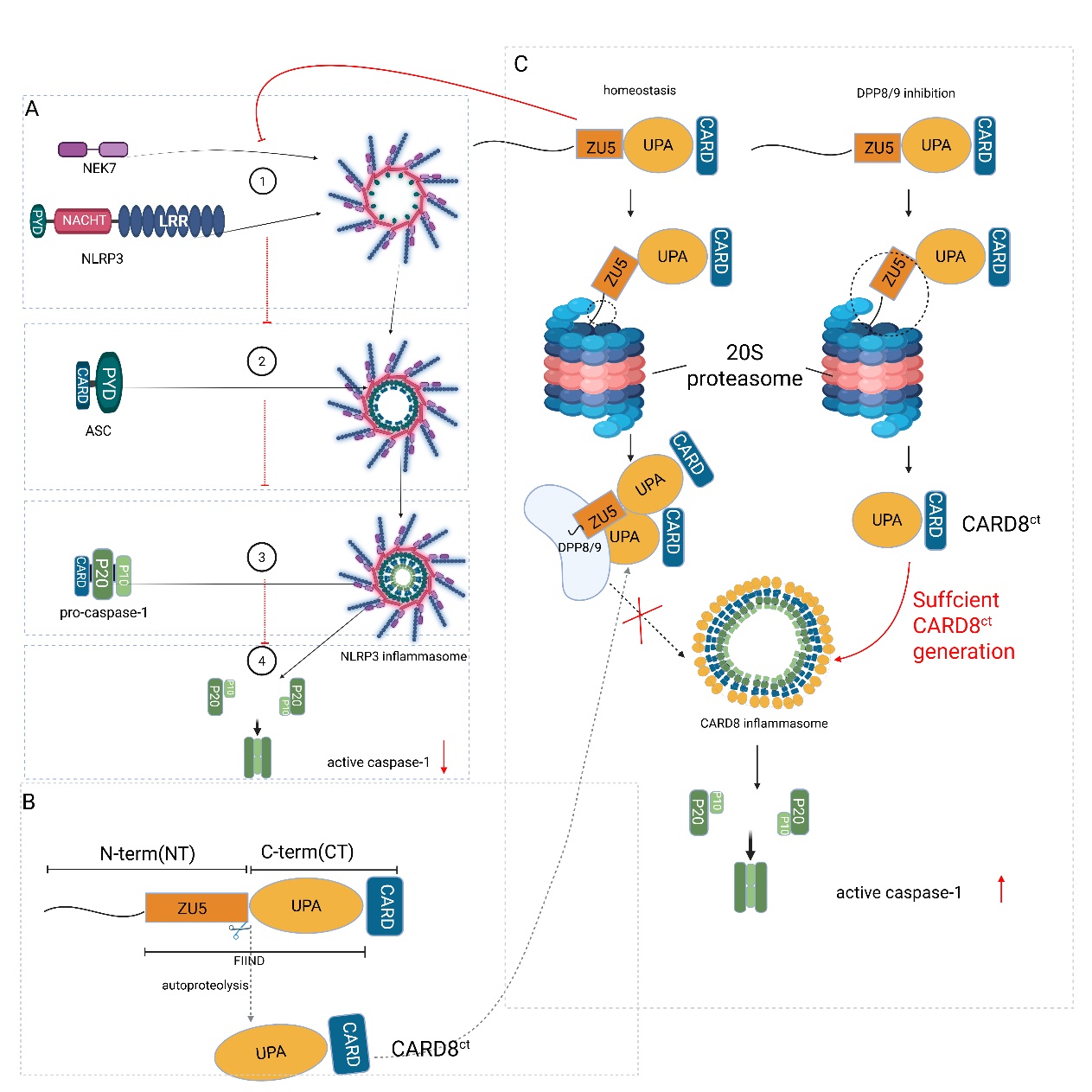


**Supplementary Figure 1. Dual role of CARD8 in regulating caspase-1 under homeostatic versus DPP8/9-inhibited conditions.**

In the absence of DPP8/9 inhibition, full-length CARD8 can normally bind to the NLRP3 domain (1) and/or the pro-caspase-1 domain (2). This can disrupt the NLRP3 inflammasome assembly and activation, leading to lower levels of active caspase-1. On the other hand, when DPP8/9 is inhibited, there is an over abundance of the CARD8^ct^ fragment, which therefore activates the CARD8 inflammasome and increases the quantities of active caspase-1.

**Figure A** represents the canonical assembly and activation process of the NLRP3 inflammasome, which includes four steps: ① NLRP3 activation and oligomerization ② Recruitment of ASC adaptor protein ③ Recruitment of pro-caspase-1 ④ Caspase-1 activation (3).

**Figure B** depicts the domain organization of CARD8, which consists of an N-terminal ZU5 domain and a C-terminal region including UPA and CARD. The FIIND region is auto-proteolytic (4), resulting in an N-terminal fragment and a C-terminal UPA-CARD fragment (CARD8^ct^). Normal conditions produce inadequate amounts of free CARD8^ct^ to activate inflammasomes (5).

**Figure C** illustrates the distinct fates of CARD8 under homeostatic conditions versus DPP8/9 inhibition. Under homeostasis, the N-terminal disordered region of CARD8 is trimmed by the 20S proteasome (6). The resulting full-length CARD8, together with the naturally occurring CARD8^ct^ fragment, forms a stable ternary complex through interaction with DPP8/9. This prevents further cleavage of the ZU5 domain and restricts excessive production of CARD8^ct^, thereby blocking inflammasome activation. In contrast, when DPP8/9 is inhibited, this protective effect on the N-terminus is lost. As a result, proteasomal cleavage extends from the disordered region into the ZU5 domain, leading to abundant release and accumulation of CARD8^ct^, which subsequently assembles the CARD8 inflammasome and activates caspase-1(6; 7).

**References:**

| 1. | Ito S, Hara Y, Kubota T. CARD8 is a negative regulator for NLRP3 inflammasome, but mutant NLRP3 in cryopyrin-associated periodic syndromes escapes the restriction. *Arthritis Res Ther* (2014) 16:R52–R52. doi: 10.1186/ar4483 |
| --- | --- |
| 2. | Razmara M, Srinivasula SM, Wang L, Poyet J-L, Geddes BJ, DiStefano PS, et al. CARD-8 Protein, a New CARD Family Member That Regulates Caspase-1 Activation and Apoptosis. *J Biol Chem* (2002) 277:13952–13958. doi: 10.1074/jbc.m107811200 |
| 3. | Fu J, Wu H. Structural Mechanisms of NLRP3 Inflammasome Assembly and Activation. *Annu Rev Immunol* (2023) 41:301–316. doi: 10.1146/annurev-immunol-081022-021207 |
| 4. | D’Osualdo A, Weichenberger CX, Wagner RN, Godzik A, Wooley J, Reed JC. CARD8 and NLRP1 Undergo Autoproteolytic Processing through a ZU5-Like Domain. *PLoS One* (2011) 6:e27396–e27396. doi: 10.1371/journal.pone.0027396 |
| 5. | Chui AJ, Griswold AR, Taabazuing CY, Orth EL, Gai K, Rao SD, et al. Activation of the CARD8 Inflammasome Requires a Disordered Region. *Cell Rep* (2020) 33:108264–108264. doi: 10.1016/j.celrep.2020.108264 |
| 6. | Hsiao JC, Neugroschl AR, Chui AJ, Taabazuing CY, Griswold AR, Wang Q, et al. A ubiquitin-independent proteasome pathway controls activation of the CARD8 inflammasome. *J Biol Chem* (2022) 298:102032–102032. doi: 10.1016/j.jbc.2022.102032 |
| 7. | Sharif H, Hollingsworth LR, Griswold AR, Hsiao JC, Wang Q, Bachovchin DA, et al. Dipeptidyl peptidase 9 sets a threshold for CARD8 inflammasome formation by sequestering its active C-terminal fragment. *Immunity* (2021) 54:1392-1404.e10. doi: 10.1016/j.immuni.2021.04.024 |


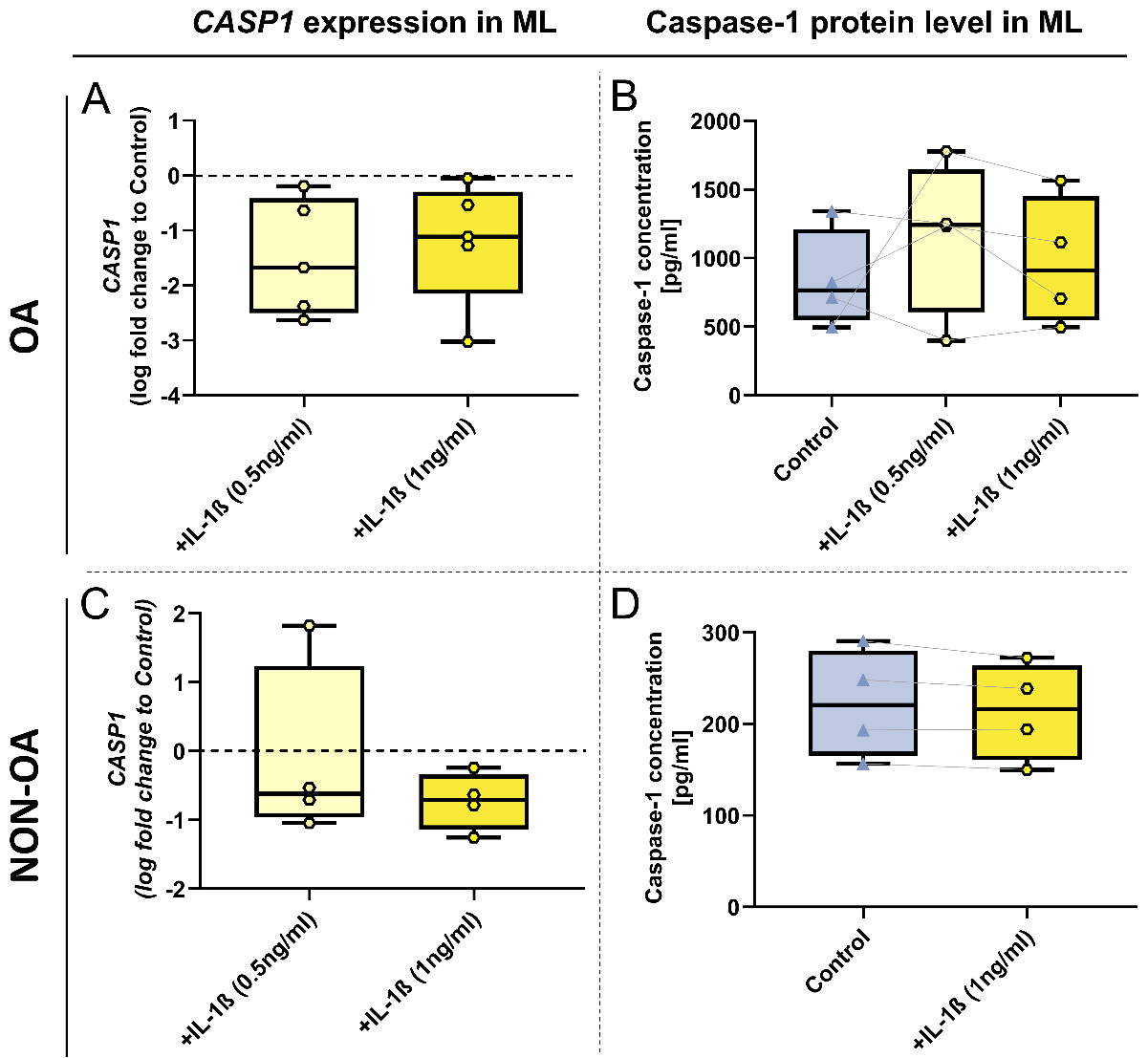


**Supplementary Figure 2. Caspase-1 mRNA expression and cell-lysate protein levels in human chondrocytes in 2D cultures following IL-1β treatment.**

A, C) CASP1 mRNA in osteoarthritic chondrocytes (OA, A) and non-osteoarthritic chondrocytes (non-OA, B) after exposure to IL-1β (0.5 ng/mL; 1 ng/mL, green). Data are shown as log2-fold change relative to untreated control (dashed line = 0). P-values were determined by one-sample t-tests against a hypothetical mean of zero. Individual symbols represent independent donors (n = 4-5).

B, D) Caspase-1 protein in OA- and non-OA cell lysates was measured by ELISA and is expressed in pg/ml. P-values were determined by one-way ANOVA with Holm–Sidak post hoc correction comparing each IL-1β group to control (n=4).


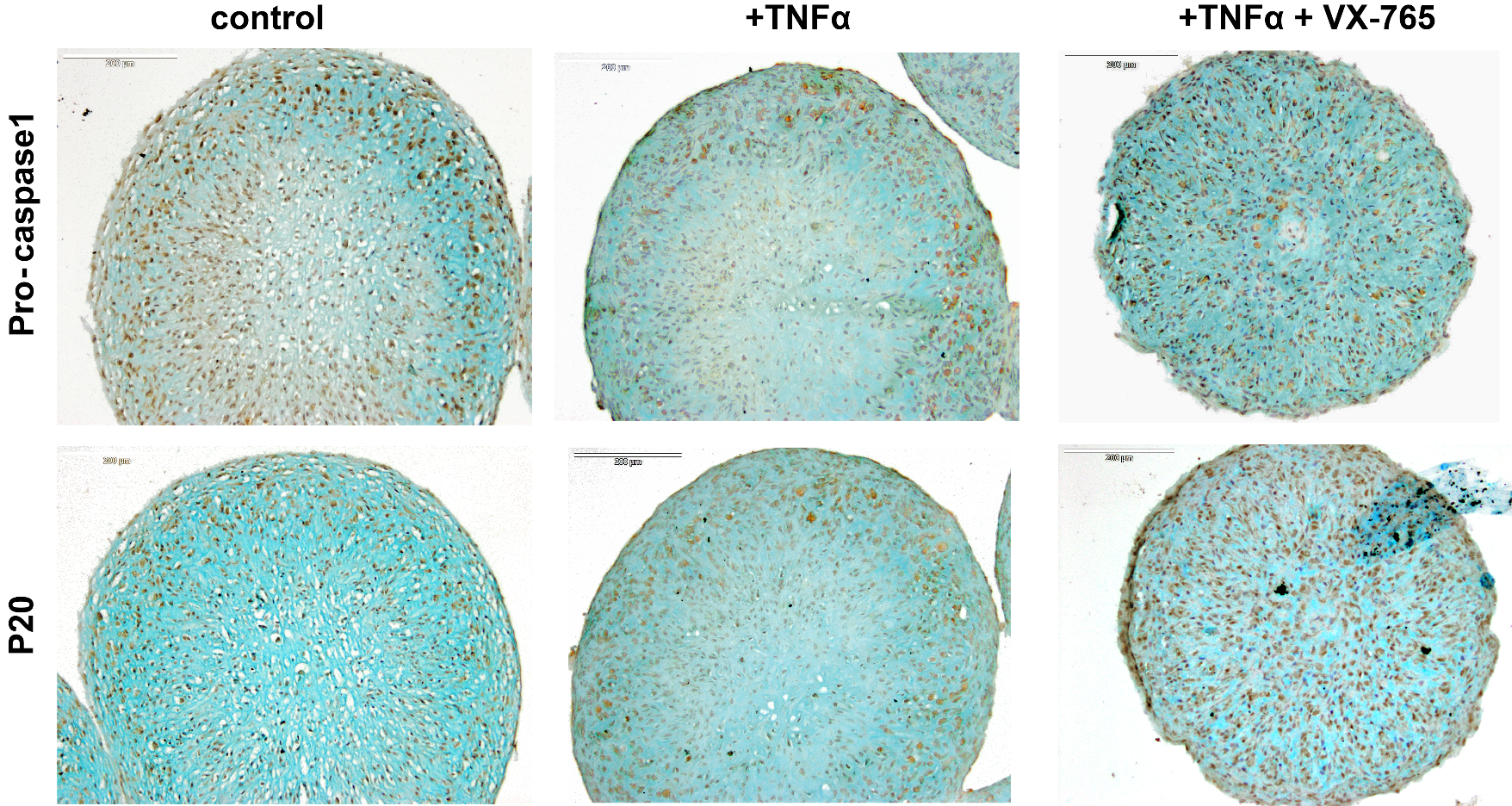


**Supplementary Figure 3. Representative immunohistochemical localization of pro- and activated caspase-1 in OA chondrocyte pellets**

Immunohistochemical staining illustrating spatial distribution patterns of pro-caspase-1 and activated caspase-1 (P20) within OA chondrocyte micromass pellets under unstimulated (control), TNF-α-stimulated (1 ng/mL), and TNF-α combined with VX-765 treatment conditions. Scale bars represent 200 µm.

**
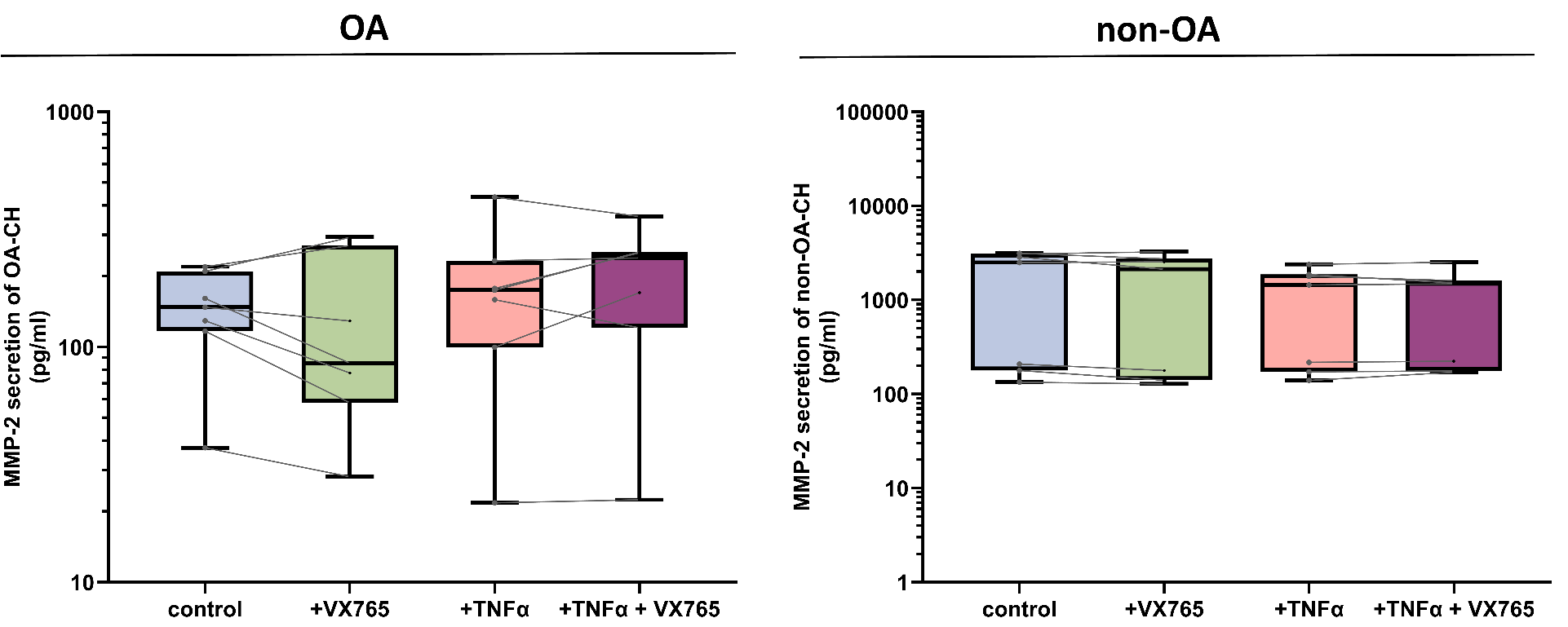
**

**Supplementary Figure 4. Quantification of MMP-2 secretion in OA- and non-OA chondrocytes after treatment with VX-765 and/or TNF-α.**

MMP-2 levels in culture supernatants of OA chondrocytes (OA-CH) and non-OA chondrocytes (non-OA CH) following treatment with VX-765 (100 µM), TNF-α (1 ng/mL) or their combination.

Concentrations were determined by Luminex-ELISA and are expressed in pg/ml. Box-and-whisker plots depict median, interquartile range and minimum/maximum; individual symbols represent independent donors (n = 6).Two-way ANOVA with Geisser-Greenhouse correction followed by Šídák's multiple comparisons test was performed.


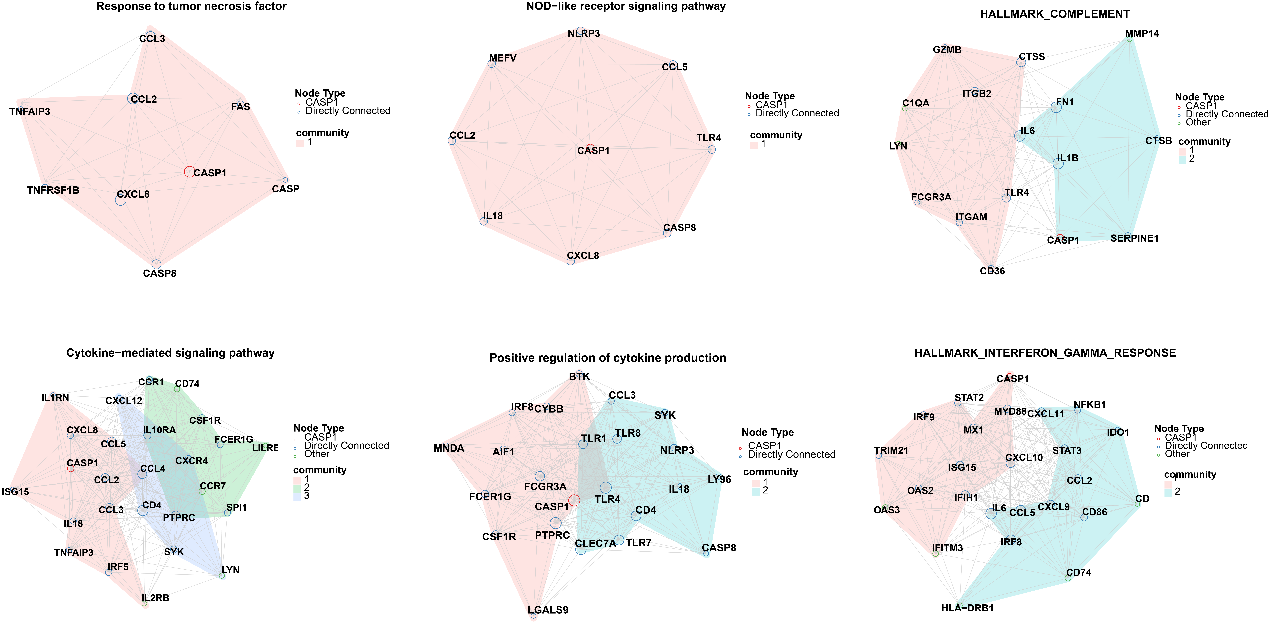


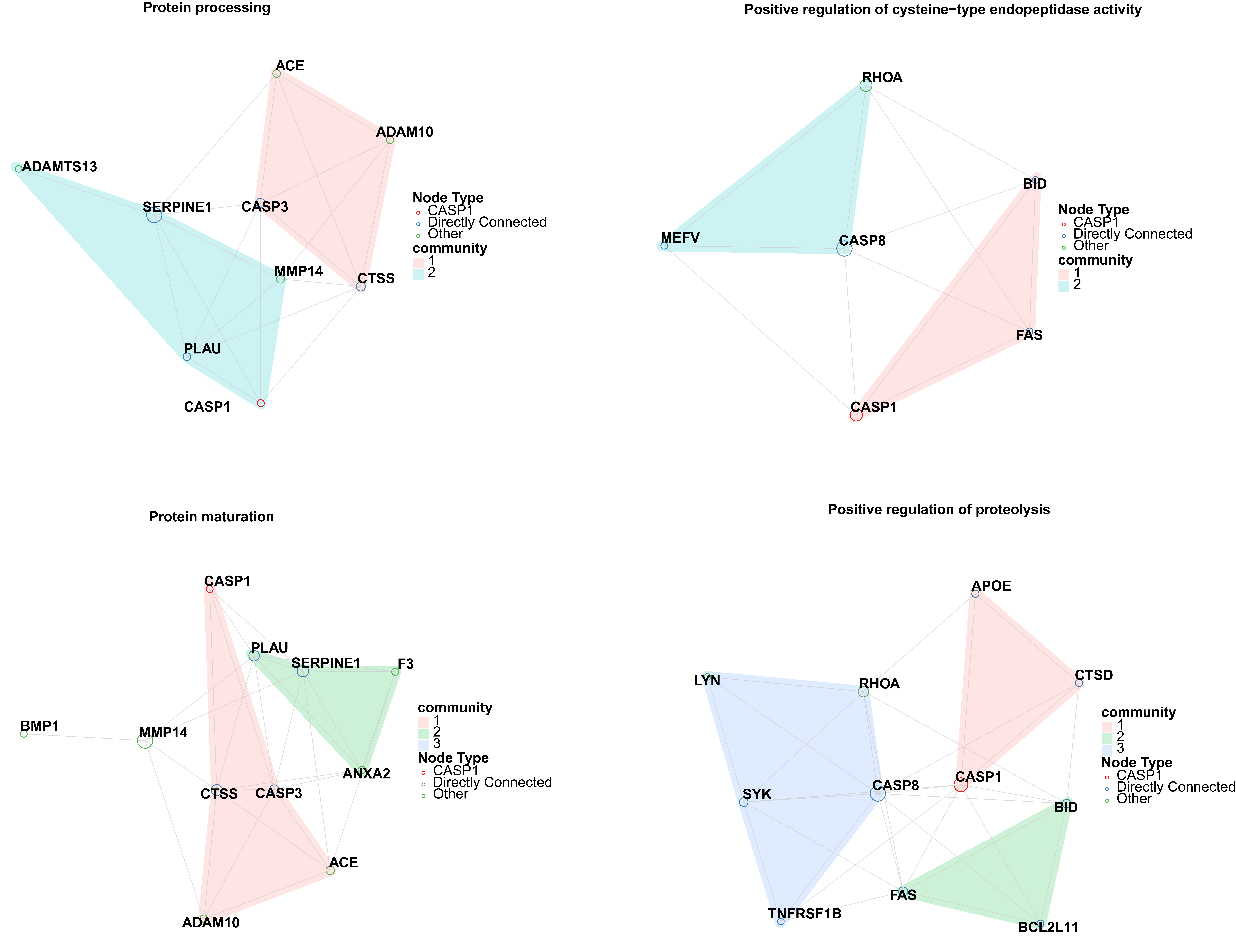

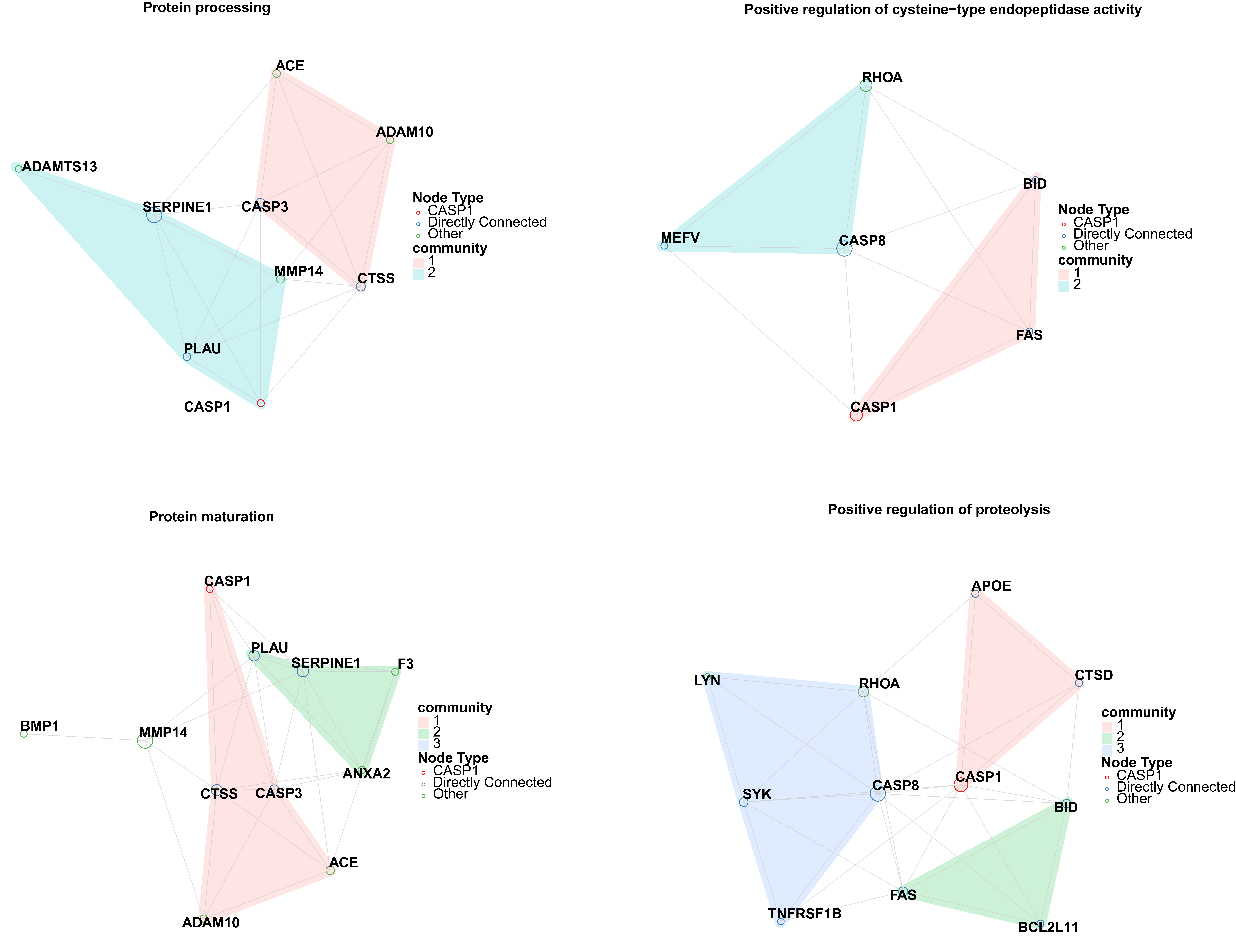


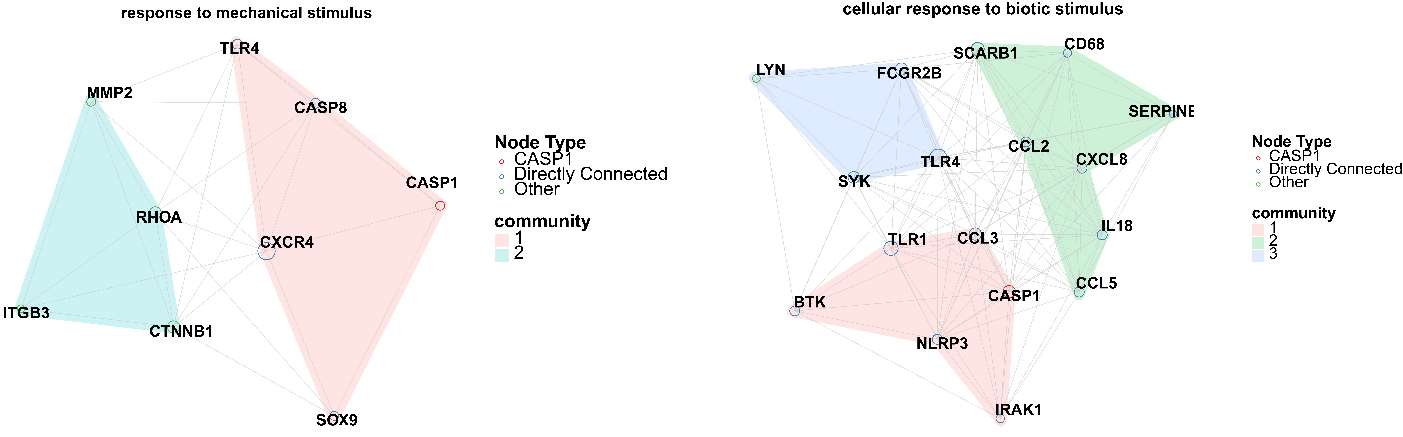


**Supplementary Figure 5. Hub-gene identification in Caspase-1–related PPI networks across functional modules.**

A) Cytokine and inflammation–associated pathways. Protein–protein interaction (PPI) networks were constructed for genes enriched in “response to tumor necrosis factor,” “NOD-like receptor signaling,” “cytokine-mediated signaling,” “positive regulation of cytokine production,” “complement,” and “interferon-gamma response” modules. Caspase-1 (square node) and directly connected interactors are shown; network communities (color-shaded clusters) represent distinct submodules.

B) Protein-processing pathways. Networks for “protein processing” and “positive regulation of cysteine-type endopeptidase activity” illustrate Caspase-1 clustering with configuration factors, proteases, and inhibitors.

C) Stress-response pathways. PPI networks for “response to mechanical stimulus” and “cellular response to biotic stimulus” highlight Caspase-1’s direct interactors within mechanotransduction and biotic-stress modules.


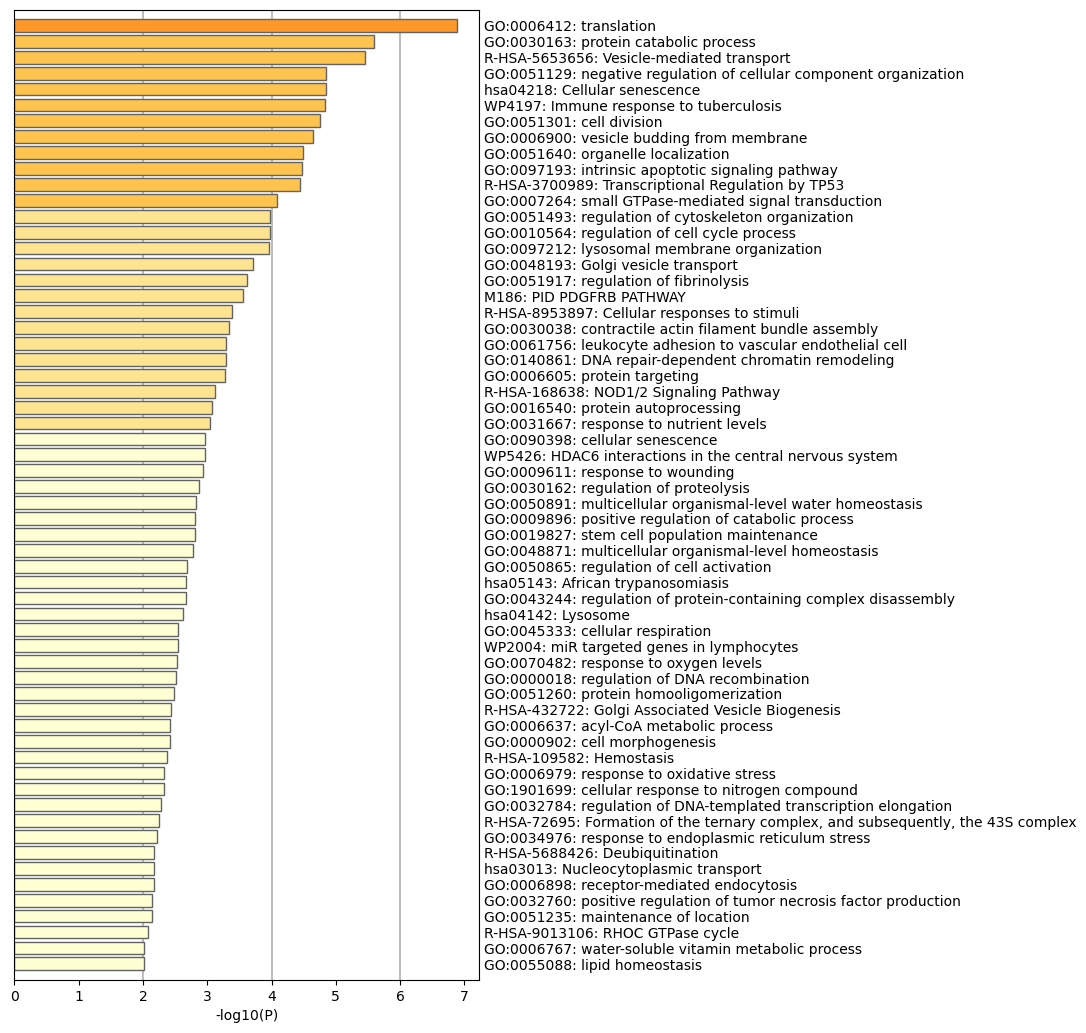


**Supplementary Figure 6. Over-representation analysis (ORA) of VX-765–regulated proteins in OA chondrocytes.**

The top 100 enriched GO and pathway terms (–log10; P ≥ 2.0) are plotted by significance. Major functional categories include protein homeostasis (translation, protein catabolic process), vesicle-mediated transport and Golgi vesicle biogenesis, cellular senescence and cell-cycle control, intrinsic apoptotic signaling and TP53-mediated transcription, NOD1/2 and TNF inflammatory signaling, cytoskeletal organization and RHO GTPase cycle, and stress-response/metabolic processes (oxidative/ER stress, lipid and vitamin metabolism).


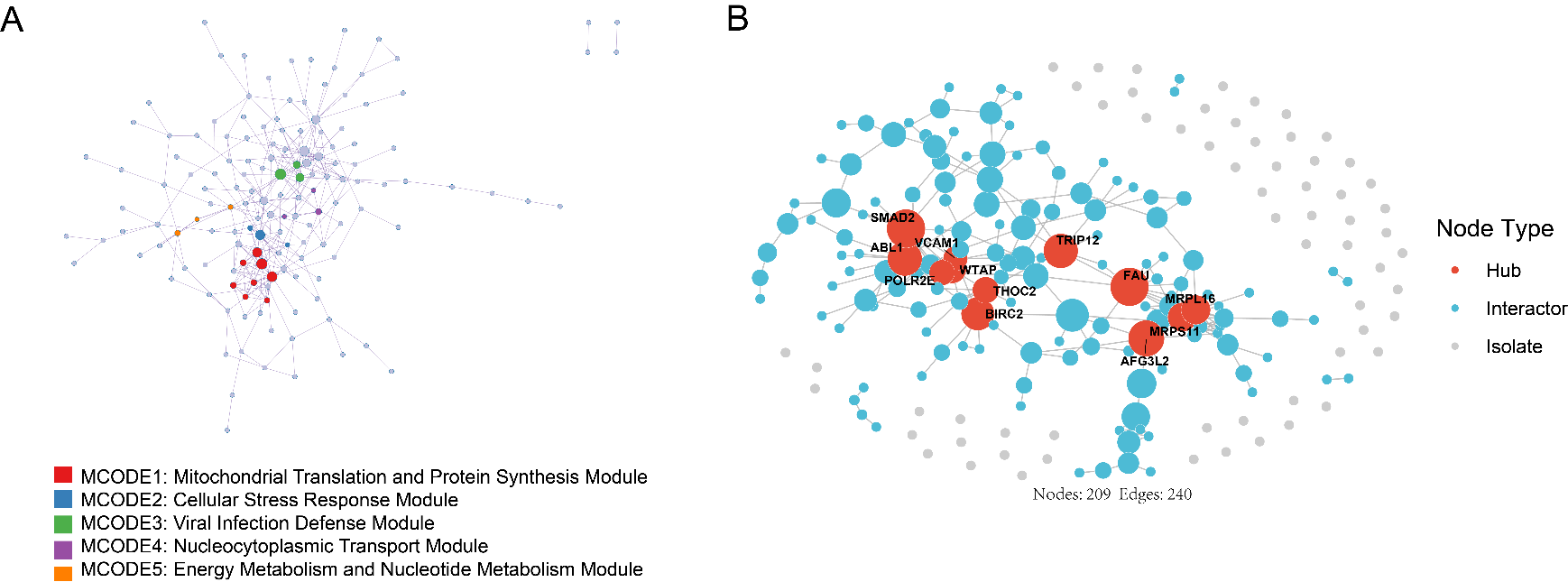


**Supplementary Figure 7. Protein-Protein- Interaction (PPI) network and hub‐gene analysis of VX-765–regulated proteins.**

A) The STRING‐derived PPI network for 209 differentially expressed proteins was clustered by MCODE into five core modules: M1 (red) mitochondrial translation and protein synthesis; M2 (blue) cellular stress response; M3 (green) viral‐infection defense; M4 (purple) nucleocytoplasmic transport; and M5 (orange) energy and nucleotide metabolism.

B) Node size reflects “betweenness” centrality; red nodes denote the 12 high-connectivity hub proteins (SMAD2, ABL1, VCAM1, WTAP, POLR2E, THOC2, BIRC2, FAU, TRIP12, MRPL16, MRPS11, AFG3L2), light blue nodes are other interactors, and grey nodes are isolates. The network comprises 209 nodes and 240 edges.


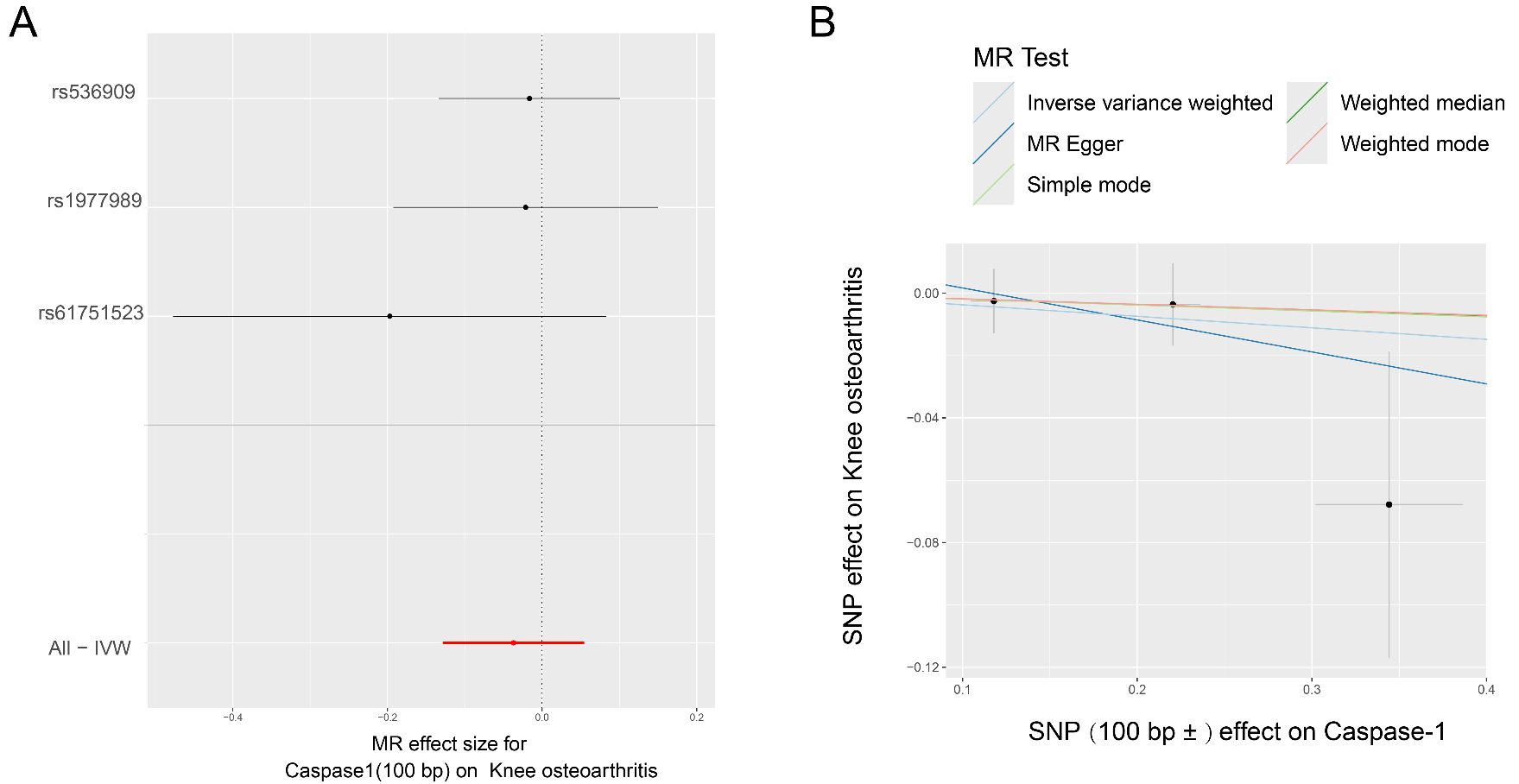


**Supplementary Figure 8. Sensitivity analysis of CASP1–OA Mendelian randomization (MR) using SNPs within ±100 bp of the CASP1 gene locus.**

A) Forest plot showing individual SNP–specific MR estimates (black dots) with 95% confidence intervals, along with the inverse‐variance weighted overall effect (red). All variants lie within 100 bp upstream or downstream of the CASP1 gene boundaries.
B) Scatter plot of each SNP’s effect on CASP1 expression (x-axis) versus its effect on knee OA risk (y-axis), with MR regression lines from IVW (blue), MR Egger (light blue), simple mode (green), weighted median (dark green) and weighted mode (pink) methods. Error bars represent standard errors of each SNP’s association estimates.


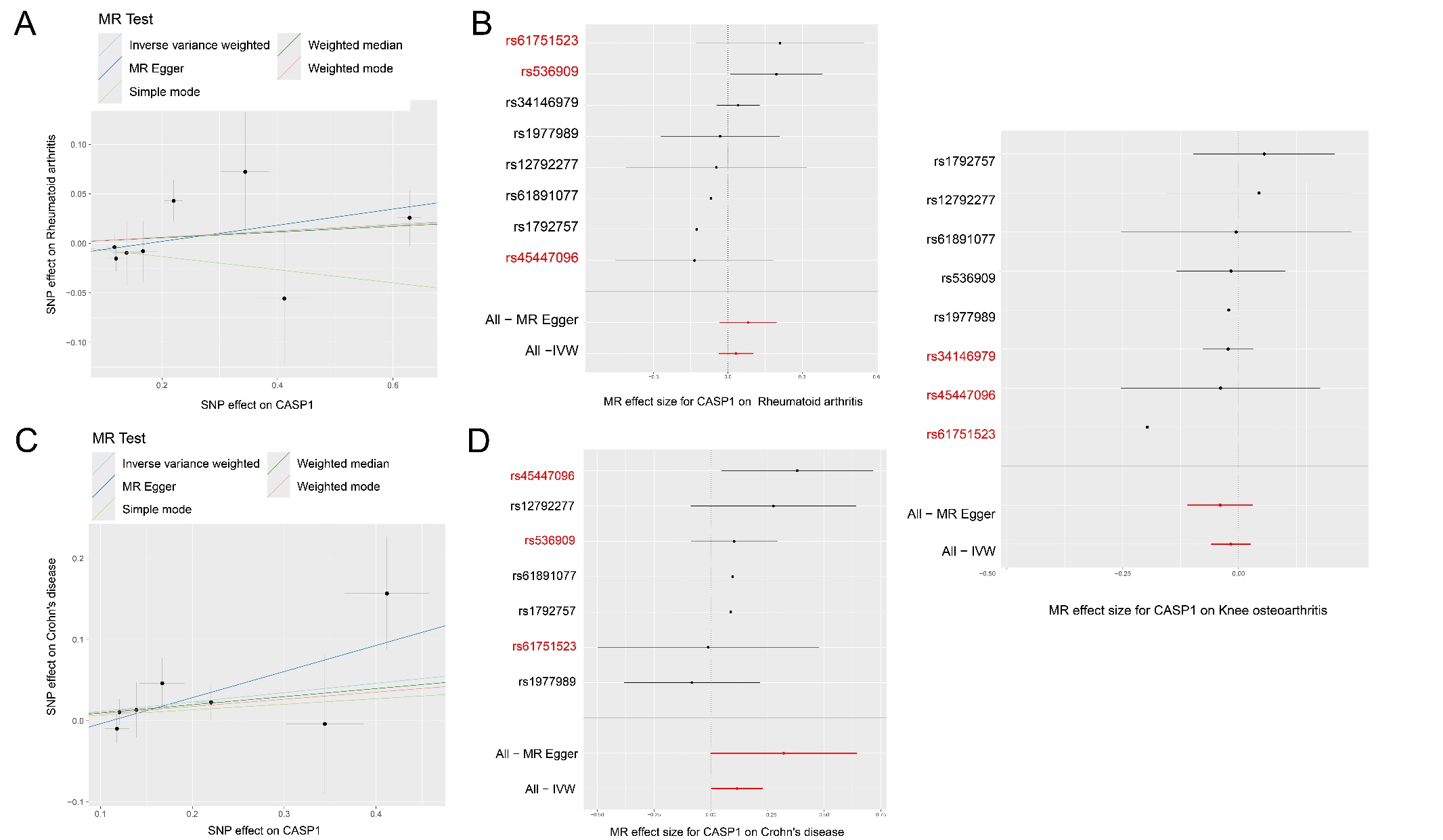
E

**Supplementary Figure** **9. Comparative Mendelian randomization of CASP1 expression on immune/inflammatory diseases versus OA.**

Panels A and C show scatter plots of SNP effect sizes on CASP1 expression (x-axis) versus their effects on rheumatoid arthritis (A) and Crohn’s disease (C) risk (y-axis). Lines depict MR estimates by inverse-variance weighted (IVW, blue), MR-Egger (green), simple mode (olive), weighted median (dark green) and weighted mode (red) methods.

Panels B, D and E are forest plots of per-SNP Wald estimates (black points ± 95% CI) and aggregate MR estimates (red diamonds) for rheumatoid arthritis (B), Crohn’s disease (D) and knee osteoarthritis (E). MR effect sizes represent log-odds ratios per unit increase in CASP1 expression.

**
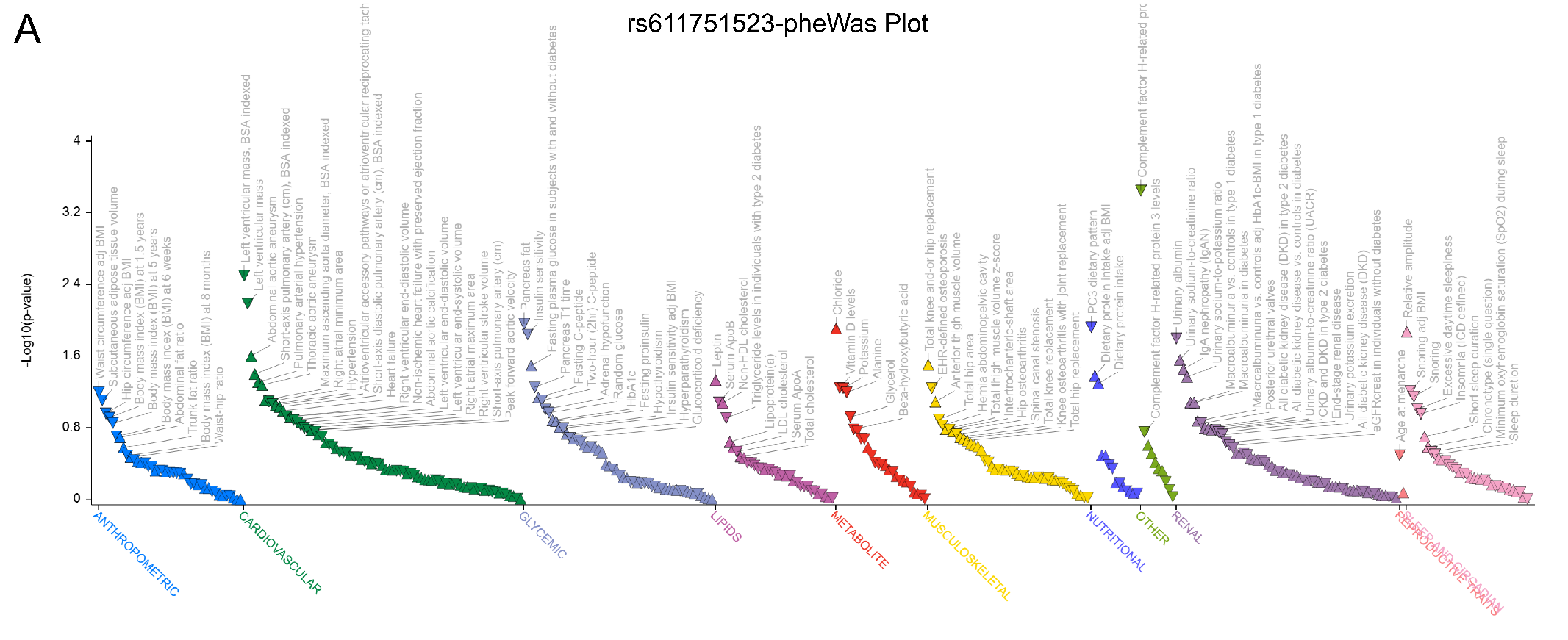
**

**
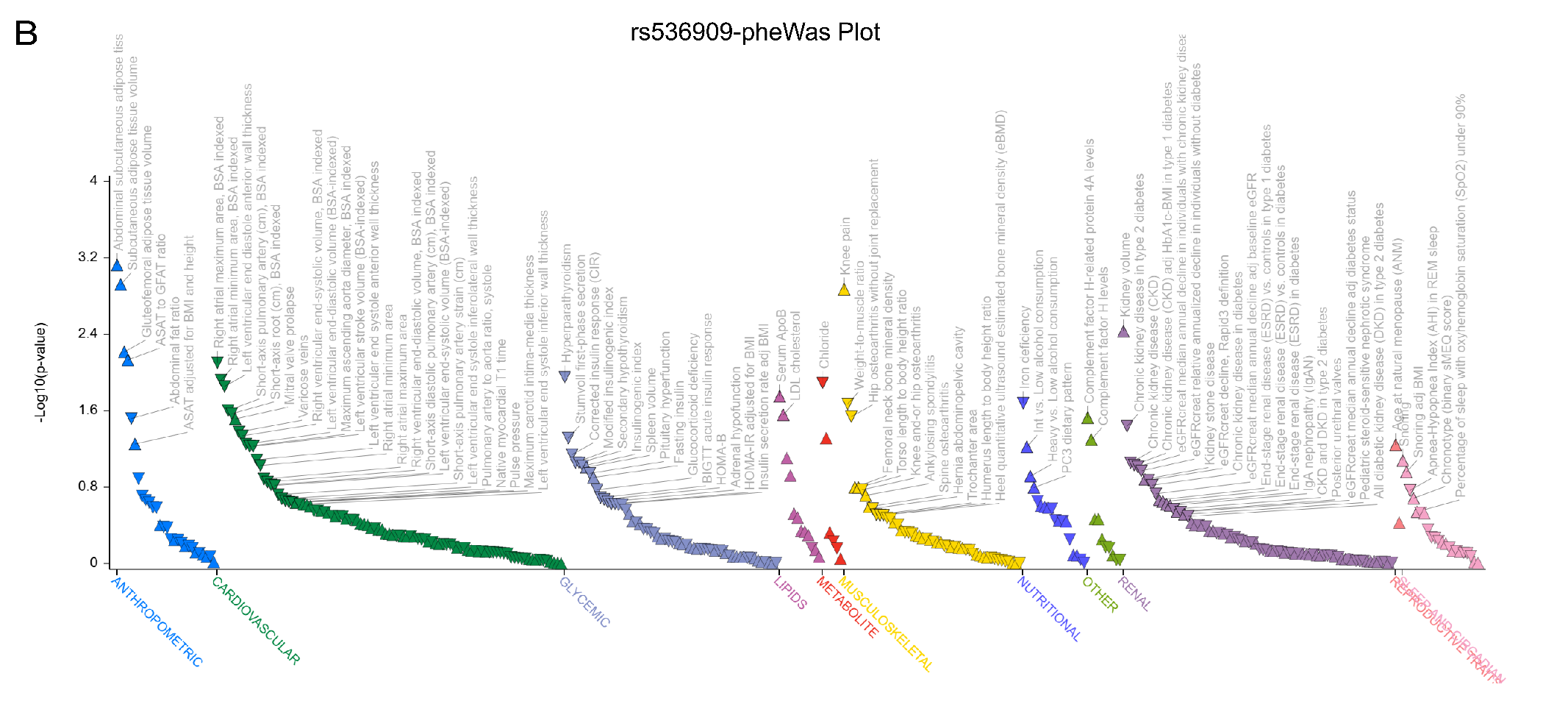
**

**Supplementary Figure 10. Phenome-wide association study (PheWAS) of CASP1 cis-eQTL variants.**

A) PheWAS plot for rs61751523.

B) PheWAS plot for rs536909

Each point represents a tested association between the SNP and a clinical or biochemical phenotype, grouped along the x-axis by trait category (color-coded). The y-axis shows the –log10 (P-value) for each SNP–phenotype test. Triangles pointing upward indicate a positive effect size, and triangles pointing downward indicate a negative effect size. The horizontal dashed line marks the phenome-wide significance threshold (P = 5×10⁻^8^; –log10P ≈ 7.3). Categories (and their colors) are: anthropometric, cardiovascular, glycemic, lipids, metabolite, musculoskeletal, nutritional, other, renal, and reproductive traits.
