# Supplementary material for: Targeting Caspase-1 in osteoarthritis: multi-omics insights into the effects of VX-765 on human chondrocyte function and phenotype
Source: Front Immunol. 2025 Oct 3;16:1677801. doi: 10.3389/fimmu.2025.1677801 (PMC12532135; doi:10.3389/fimmu.2025.1677801)
Supplement: Supplementary file 2 [file DataSheet2.docx]

Targeting Caspase-1 in Osteoarthritis: Multi-Omics Insights into the Effects of VX-765 on Human Chondrocyte Function and Phenotype

Jian Mei, Nicole Schäfer, Penghui Wei, Zhiheng Kong, Shushan Li, Patrick Pann, Marianne Ehrnsperger, Brian Johnstone, Eva Matalova , Susanne Grässel#

**Supplementary Methodology**

Note: This section provides a detailed supplement to the omics data analysis procedures, intended to support interpretation and reproducibility. The corresponding methods were briefly described in the main text and are elaborated here following the order of the result presentation.

**1. Characterization of Caspase-1 Expression in OA versus non-OA Chondrocytes ( mRNA sequence analysis of GSE16850)**

**1.1 Differential Gene Expression Analysis**

Raw RNA-seq counts for primary human chondrocytes (GSE168505) were imported and annotated to HGNC symbols using org.Hs.eg.db in R (v4.2.2). Genes without mapping were removed, and duplicates were resolved by unique suffixes. Differential expression analysis was performed with DESeq2 (OA, n = 4; non-OA, n = 3), using default normalization and Wald tests. Genes with adjusted p < 0.05 and |log₂FC| > 0.9 were considered significant. Volcano plots were generated with ggplot2, highlighting key inflammasome, senescence, and cartilage degradation markers.

**1.2 Gene Expression Correlation**

CASP1 expression was correlated with selected gene sets (SenMayo, Hallmark Inflammatory Response, Hallmark Oxidative Phosphorylation, Reactome Extracellular Matrix, Reactome Cell Cycle) using log₂-transformed, normalized RNA-seq data. Low-variance genes were excluded. Pearson correlations were computed with Hmisc (rcorr), and p-values were FDR-adjusted.

**2. Transcriptome and proteomics reveal a multilevel pathological role of Caspase-1 in OA**

**2.1. Transcriptome-based functional enrichment analysis and hub-gene identify**

**2.1.1 GO and KEGG Enrichment Analysis with Functional Categorization**

DEGs between OA and non-OA chondrocytes (|log₂FC| > 0.9, adjusted p < 0.05) were analyzed with clusterProfiler (v4.2.2) in R. GO enrichment (BP, MF, CC) and KEGG pathways (organism = "hsa") were tested with Benjamini–Hochberg correction. Enriched terms related to genes of interest (e.g., CASP1) were grouped into functional categories (e.g., Inflammation, Cell Death, Metabolism) and visualized as bubble plots in ggplot2.

**2.1.2 GSEA Analysis with Functional Categorization**

Log₂ fold change–ranked gene lists (DESeq2 output) were analyzed with clusterProfiler (fgsea algorithm, R) using Hallmark gene sets (MSigDB v7.4). Pathways with adjusted p < 0.05 were considered significant. To assess the role of CASP1, significant pathways containing CASP1 in the leading-edge subset were grouped into Inflammation & Immunity or Cell Death & Stress Response and visualized with enrichment curves.

**2.1.3 Protein-Protein Interaction Network Construction and Hub Gene Identification of OA-related genes of bulk-mRNA sequencing data**

PPI networks for CASP1-associated pathways were constructed using the STRING database (https://string-db.org/, confidence ≥ 0.4, all active sources). Resulting interaction files were processed in R (igraph, tidygraph, ggraph) to build undirected graphs and calculate centrality metrics (degree, closeness, betweenness). Genes scoring above the mean across all three metrics were defined as hub genes.

CASP1-centered subnetworks were then extracted by retaining hub genes and their direct interactors with CASP1. Networks were visualized using force-directed layouts with community clustering, and exported in multiple formats for downstream analysis.

**2.2 Proteomics-based validation and extended analysis**

**2.2.1 Differential protein analysis**

DIA-based quantitative proteomics data (Orbitrap Astral) were processed with DIA-NN against the UniProt-SwissProt human database (release 2024-07-26). Protein-level Q-values were filtered at < 0.01. For each group comparison, fold change (FC) was calculated from mean protein abundances, and significance was assessed using two-tailed Student’s t-tests. Differentially expressed proteins (DEPs) were defined as FC > 1.2 or < 0.83 with p < 0.05.

DEPs were visualized with volcano plots to assess expression profiles and group variance.

**2.2.2 Subcellular Localization**

Protein subcellular localization was predicted using the Cell-mPLoc 2.0 database. Predicted sites were grouped into major compartments (nucleus, cytoplasm, mitochondria, plasma membrane, extracellular region, endoplasmic reticulum, Golgi apparatus), and their distributions were visualized with pie charts.

**2.2.3 ORA of Differential Proteins**

Differentially expressed proteins (DEPs) were analyzed in Metascape (https://metascape.org

) across multiple ontology sources (GO Biological Processes, KEGG, Reactome, Canonical Pathways, CORUM, WikiPathways), using the full human genome as background. Enriched terms (p < 0.01, gene count ≥ 3, enrichment factor > 1.5) were clustered by functional similarity, with representative terms from each cluster retained. Top enriched terms were visualized as bar plots of –log₁₀(p).

**2.2.4 Protein–Protein Interaction (PPI) Network and Module Analysis of Differential Proteins**

**Compilation of Differentially Expressed Proteins**

A total of 209 differentially expressed proteins (DEPs) after VX-765 treatment were analyzed using the STRING database (v11.5, confidence ≥ 0.4). The resulting undirected network was processed in R (igraph, tidygraph) to calculate degree, closeness, and betweenness centralities. Proteins exceeding mean values across all three metrics were defined as hub genes. Functional modules were further identified with the MCODE algorithm in Metascape, yielding clusters related to mitochondrial translation, stress response, antiviral defense, nucleocytoplasmic transport, and energy/nucleotide metabolism.

**2.2.5 Molecular docking analysis of Caspase-1 and key downstream molecules**

Crystal structures of Caspase-1 and candidate interactors (e.g., ABL1) were retrieved from the Protein Data Bank (PDB). For Caspase-1, the activated heterodimer structure (p20 and p10 subunits; PDB ID: 6BZ9) was selected. The structure was pre-processed in PyMOL by removing crystallographic water molecules, the covalently bound inhibitor (Ac-YVAD-CMK, chain C), and non-target chains, retaining only chains A (p20) and B (p10) to represent the active form of Caspase-1. Similarly, for the interacting proteins, non-essential ligands and water molecules were removed, and only the target protein chains were retained. Docking was performed with the GRAMM-X server (Caspase-1 as receptor), and the top-ranked pose was selected. Interfaces were evaluated using PDBePISA to assess contacts, buried surface area, and binding free energy (ΔG). Complexes were visualized in PyMOL, highlighting interface residues and key interactions.

**2.2.6 GSEA of Proteome-Wide Expression Changes**

Protein-level log₂ fold changes were ranked and analyzed with the clusterProfiler package (fgsea algorithm, R v4.2.2). Gene sets included GO (BP, MF, CC), KEGG, MSigDB Hallmark (v7.4), the senescence-associated SenMayo signature, and InterPro protein domains (downloaded from the EMBL-EBI InterPro database). Enrichment was considered significant at adjusted p < 0.05. GO, KEGG, and domain terms were grouped into functional categories (e.g., Cytoskeleton, Metabolism, Immune Response) and visualized as grouped bar plots, whereas Hallmark and SenMayo enrichment results were displayed with enrichment curves.

**2.2.7 Cross-omics validation**

To compare pathway changes across omics layers, GSEA was performed separately on bulk RNA-seq (OA vs. non-OA chondrocytes) and proteomics data (VX-765–treated vs. untreated OA chondrocytes) using clusterProfiler with Hallmark and SenMayo gene sets. Gene/protein lists were ranked by log₂ fold change, and pathways with adjusted p < 0.05 were considered significant.

Pathways enriched in both datasets were identified, and their normalized enrichment scores (NES) were compared to assess whether VX-765 reinforced or opposed OA-associated alterations. Shared pathways were visualized in paired plots for direct comparison.

**2.3 Mendelian Randomization (MR) Analysis of caspase-1 and its Regulatory factors on OA.**

Study Design and Data Sources

Two-sample Mendelian randomization (MR) was performed to assess potential causal effects of genetically predicted expression of CASP1, CARD8, CARD17, and CARD18 on osteoarthritis (OA) risk. Cis-eQTLs (P < 5 × 10⁻⁸, within ±100 kb of each gene) were obtained from the eQTLGen Consortium (whole blood, European ancestry). OA GWAS summary statistics were derived from the GO Consortium and UK Biobank meta-analysis (GCST007090; 24,955 knee OA cases, 15,704 hip OA cases, 378,169 controls, European ancestry). All datasets were publicly available and ethically approved by their original studies (see Supplementary Table 4).

Genetic Instrument Selection

Instrumental variables (IVs) were selected using the following criteria:

(1) Statistical significance: SNPs associated with gene expression at genome-wide significance (P < 5 × 10⁻⁸). (2) Minor allele frequency: SNPs with MAF > 0.01 were retained. (3) LD clumping: Variants in linkage disequilibrium were pruned using a clumping window of 100 kb and an LD threshold of R² < 0.3, based on the 1000 Genomes European reference panel. The SNP with the smallest P-value in each LD block was retained. (4) Allele harmonization: Exposure and outcome datasets were aligned to ensure consistent effect allele orientation. Palindromic and ambiguous SNPs were excluded.

MR Estimation and Sensitivity Analyses

Causal effects were estimated using inverse-variance weighted (IVW) regression under a random-effects model. Robustness was assessed by sensitivity analyses, including MR-Egger (pleiotropy), Cochran’s Q (heterogeneity), weighted median/mode estimators, single-SNP analysis, and Steiger filtering. Results were expressed as odds ratios (ORs) with 95% confidence intervals per standard deviation increase in genetically predicted expression. Analyses were conducted with TwoSampleMR (v0.5.6) in R 4.2.2..

Specificity Analysis

To evaluate specificity, the same CASP1-related instruments were tested against GWAS data for rheumatoid arthritis and Crohn’s disease. Analyses focused on the direction and consistency of SNP effects rather than effect size. Dataset details are provided in Supplementary Table 4.

**2.4 Cross-Trait Pleiotropy and Motif Disruption Analysis**

Selected SNPs (e.g., rs580253) were queried in the MSK-HuGEAMP platform (https://msk.hugeamp.org/) to examine associations with diverse traits and assess potential disruption of transcription factor binding motifs.
